# Educational Needs in Geriatric Medicine Among Health Care Professionals and Medical Students in COST Action 21122 PROGRAMMING: Mixed-Methods Survey Protocol

**DOI:** 10.2196/64985

**Published:** 2025-06-03

**Authors:** Giulia Ogliari, Tahir Masud, Anna Marie Herghelegiu, Tajana Pavic, Anne Wissendorff Ekdahl, Karolina Piotrowicz, Sofia Duque, Athanase Benetos, Nenad Bogdanovic, Sylvie Bonin-Guillaume, Rachael Frost, Meltem Koca, Anastassia Kossioni, Evrydiki Kravvariti, Nicolas Martínez-Velilla, William McKeown, Regina Roller-Wirnsberger, Sumru Savas, Michael Vassallo, Tamar Yellon, Mirko Petrovic, Marina Kotsani, Collaborators of the PROGRAMMING survey on educational needs

**Affiliations:** 1 Department of Health Care of Older People, Queen's Medical Centre Nottingham University Hospitals NHS Trust Nottingham United Kingdom; 2 Department of Internal Medicine Faculty of Medicine Carol Davila University of Medicine and Pharmacy Bucharest Romania; 3 Ana Aslan National Institute of Gerontology and Geriatrics Bucharest Romania; 4 Department of Internal Medicine Division of Gastroenterology and Hepatology University Hospital Center Zagreb Croatia; 5 Institution of Clinical Sciences Helsingborg Faculty of Medicine Lund University Lund Sweden; 6 Department of Internal Medicine and Gerontology Faculty of Medicine Jagiellonian University Medical College Kraków Poland; 7 Hospital CUF Descobertas Lisbon Portugal; 8 Preventive Medicine and Public Health Institute Faculty of Medicine University of Lisbon Lisbon Portugal; 9 Geriatric Dpt, CHRU Nancy and INSERM DCAC Université de Lorraine Nancy France; 10 Department of Geriatric Medicine Karolinska University Hospital and Karolinska Institutet Stockholm Sweden; 11 Aix Marseille University University Hospital of Marseille Marseille France; 12 School of Public and Allied Health Liverpool John Moores University Liverpool United Kingdom; 13 Turkish Republic Ministry of Health Etlik City Hospital, Yenimahalle Ankara Turkey; 14 Dental School National and Kapodistrian University of Athens Athens Greece; 15 First Department of Propaedeutic Internal Medicine School of Medicine National and Kapodistrian University of Athens Athens Greece; 16 Navarre Health Service (SNS-O), Navarre University Hospital (HUN), Department of Geriatrics, Navarrabiomed, Navarre Public University (UPNA), Navarra Institute for Health Research (IdiSNA) Pamplona Spain; 17 Department of Care of the Elderly Antrim Area Hospital Antrim United Kingdom; 18 Department of Internal Medicine Medical University of Graz Graz Austria; 19 Section of Geriatrics, Department of Internal Medicine School of Medicine Ege University Izmir Turkey; 20 Royal Bournemouth Hospital University Hospitals Dorset Bournemouth United Kingdom; 21 The Henrietta Szold Nursing Department The Faculty of Medicine The Hebrew University of Jerusalem Jerusalem Israel; 22 Jerusalem College of Technology Jerusalem Israel; 23 Section of Geriatrics Department of Internal Medicine and Paediatrics Ghent University Ghent Belgium; 24 Hellenic Society for the Study and Research of Aging Athens Greece; 25 see Acknowledgements Nottingham United Kingdom

**Keywords:** geriatric medicine education, open online survey, questionnaire design, teaching methods, health care professionals, qualitative research, emerging geriatric medicine

## Abstract

**Background:**

The European Cooperation in Science and Technology (COST) Action 21122, PROmoting GeRiAtric Medicine in countries where it is still eMergING (PROGRAMMING) developed an online open survey to assess the educational interests and needs of health care professionals and final-year medical students across participating countries. This survey aims to establish a current baseline for developing educational content on geriatric medicine for nongeriatricians and a framework for its delivery.

**Objective:**

This paper describes the aim, development, structure, content, and dissemination of this survey.

**Methods:**

The mixed methods electronic survey, initially developed in English through a cocreation process with key stakeholders, was subsequently translated into 24 languages. It received ethics approval from multiple participating countries. Within- and cross-country analyses of the survey data will be conducted using descriptive and inferential statistics for quantitative data and content analyses for qualitative data. National and international teams will conduct analyses in parallel exploring responses within a specific country or region, professional category (or among medical students), or setting of work. Basic descriptive statistics and chi-square tests will evaluate differences in knowledge, relevance, and interest in geriatric topics across countries, professions, and settings of work. The effectiveness of formal education in geriatric medicine and clinical rotations in geriatric settings versus the lack thereof in promoting higher self-perceived knowledge on geriatric medicine topics will be explored using binary logistic regression. We will provide basic descriptive statistics (frequencies) of reported barriers to receiving further training in geriatric medicine and the effectiveness of various teaching methods as rated by the respondents and explore differences across countries, professions, and settings using chi-square tests. We will conduct qualitative content analyses of free-text responses to the questions exploring professionals’ and medical students’ thoughts on caring for older people and medical students’ thoughts on becoming geriatricians.

**Results:**

The survey included the following sections: Informed Consent, Demographics, Topics and Skills, Medical Students vs. Professionals, Current Profession (for professionals), Previous Education in Geriatric Medicine (for professionals), Education in Geriatric Medicine (for medical students), Interest in Care of Older People or Geriatric Medicine, Suggestions for Courses in Care for Older People or Geriatric Medicine, and Closure. The survey was disseminated between October 9, 2023, and June 5, 2024, and received 6099 responses; after cleaning, there were 5922 (97.1%) responses (n=5474, 92.43% from professionals and n=448, 7.57% from medical students).

**Conclusions:**

This survey’s findings will inform educational projects across the PROGRAMMING countries. We will share these findings with national and international stakeholders, including professional societies, medical schools, and other relevant organizations. We will advocate for professional educational curricula to include geriatric topics rated as relevant by the survey respondents and promote clinical rotations in geriatric settings and teaching methods rated as effective by the survey respondents.

**International Registered Report Identifier (IRRID):**

DERR1-10.2196/64985

## Introduction

### Background

The world’s population is aging, with an estimated 703 to 829 million adults aged ≥65 years, accounting for almost 10% of the global population [1,2]. In Europe, as of June 2024, there were 153 million adults aged ≥65 years, or 20.6% of the population [2]. In the last few decades, both the number and proportion of older adults have increased in most areas of the world due to increasing life expectancy and declining birth rates [1,3]. The European Union (EU) statistics showed a decline in life expectancy in 2020 after the COVID-19 pandemic compared with 2019 [4]. In 2022, the EU life expectancy at birth was 80.6 years, whereas that at the age of 65 years was 19.5 years [4,5]. Moreover, men and women aged 65 years in the EU may expect to live a further 9.5 and 9.9 healthy, disability-free years, respectively, while facing disability in the remaining years [6]. Indeed, multimorbidity and disability become more common with age [7]. The health care complexity and needs of older adults differ from those of younger adults [8,9]. Older adults experience age-related changes in their physiology and may face age-associated diseases, multimorbidity, disability, and geriatric syndromes such as frailty [8,9], which are relevant to all health care professionals participating in the care of older adults.

The needs of older adults place demands on both the health care and social welfare systems. However, there is a global shortage of health care workers, which is projected to reach 10 million health care workers by 2030, equivalent to approximately 20% of the workforce needed [10]. This shortage will occur mostly in low- and middle-income countries while also affecting the EU [11]. In 2021, there were approximately 1.82 million practicing physicians and 2.8 million nurses in the EU but with an unequal distribution across countries [11]. In particular, there is a shortage of physicians and nurses in many Eastern European countries [11]. Moreover, the health care workforce is aging across many EU countries as the baby boomer generation reaches retirement age. In 2021, nearly 40% of all physicians in the EU were aged ≥55 years [12].

This shortage is especially striking in the case of health care workers with training and expertise in the care of older adults [11]. In addition, there is significant heterogeneity in the practice and recognition of geriatric medicine as a medical specialty [13,14]. Currently, geriatric medicine is still not recognized as a medical specialty in several European countries, leading to a lack of not only clinicians but also educators.

In view of the growing aging population, every health care professional should ideally receive basic education and training in the care of older adults [15-18]. Most health care professionals will deal with older adults in their clinical practice in most clinical settings [15-18]. The care of older adults should integrate person-centered assessments of their intrinsic capacity—pertaining to all physical, sensory, cognitive, and psychological capacities of an older person—and functional ability [8,9].

Minimum geriatric competencies have been set for graduating medical students in Europe, the United States, and Japan [15,17,19]. In 2007, the Association of American Medical Colleges developed 26 minimum geriatric competencies for medical students, all of which were performance-focused competencies [17]. Later, learning objectives in geriatric mental health were proposed for medical students [18]. Moreover, core competencies and educational training requirements have been established for medical doctors specializing in geriatric medicine across Europe [20,21]. Geriatric training for dental students is included in the European College of Gerodontology undergraduate curriculum guidelines [22], whereas, recently, core competencies in oral health assessment and promotion of older adults have been established for all nondental health care professionals [23]. Further research has addressed the curricula of specialized nurses [24]. However, there is not yet a recommended set of core geriatric competencies for nongeriatrician physicians and surgeons or other health care professionals.

The European Cooperation in Science and Technology (COST) is a funding organization for research and innovation networks [25]. COST Actions help connect research initiatives across Europe and beyond and enable researchers and innovators to grow their ideas in any science and technology field by sharing them with their peers [25]. COST Actions are bottom-up networks with a duration of 4 years that boost research, innovation, and careers [25]. COST recognizes the importance of education in geriatric medicine across Europe. As a result of this and the quality of the proposal suggested, COST decided to allow funding for the PROmoting GeRiAtric Medicine in countries where it is still eMergING (PROGRAMMING) COST Action CA21122 proposal [26]. The European Geriatric Medicine Society (EuGMS) is the grant holder of PROGRAMMING [27].

PROGRAMMING CA21122 aims to reach a consensus about the basic content of geriatric educational programs for a broad range of health care professionals that would consider local contexts, needs, and assets of stakeholders and the constraints of individual settings [26]. Ideally, minimum geriatric competencies should be established for a broad range of health care professionals. We envisage further objectives that go beyond PROGRAMMING but could be facilitated by its outputs. The first is to not only to educate and train graduating health care professionals but also provide curricula for professional education for postqualified and practicing professionals in several countries. Many of these professionals have received little or no education in the care of older adults, and the few that have often require up-to-date education and training in emerging topics and skills such as deprescribing medications or managing delirium [16]. Second, we need to explore how digital education, which has been increasingly used since the COVID-19 pandemic, may promote effective, harmonized, affordable, and accessible education of health care professionals in countries with different income levels and infrastructure [28]. We hope that European countries where geriatric medicine is traditionally well established may share their experience with European countries where geriatric medicine is still emerging.

**Figure 1:**
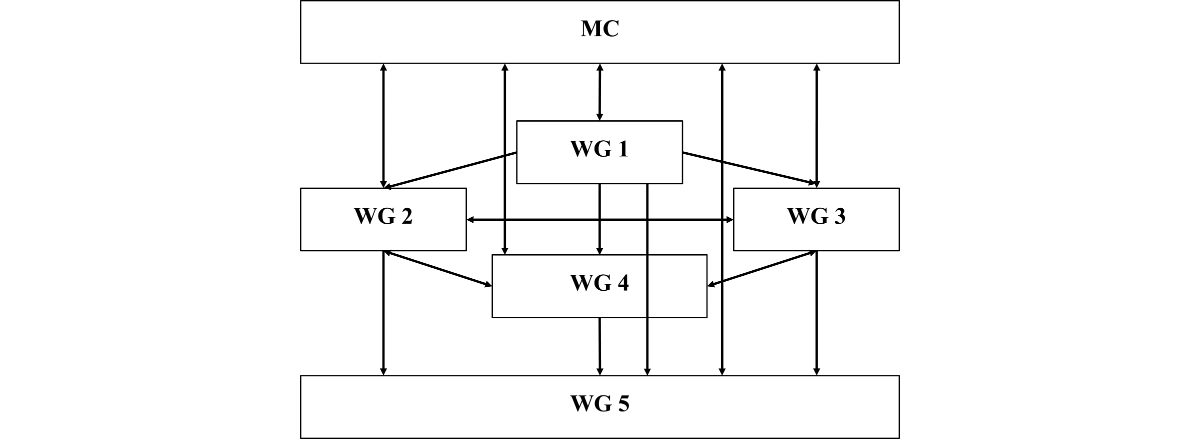
Structure of the European Cooperation in Science and Technology Action PROmoting GeRiAtric Medicine in countries where it is still eMergING (PROGRAMMING) CA21122. MC: managing committee; WG: working group.

In February 2025, PROGRAMMING included 355 participants from 43 countries [26]. It comprised a leading core group, a coordinating managing committee (MC) with representatives from most participating countries, and 5 working groups (WGs; Figure 1) [26]. WG 1 aims to map the educational needs in the field of geriatric medicine across participating countries. WG 2 and WG 3 focus on the content of training of health care professionals working in community and primary care settings (WG 2) and acute care hospitals and long-term care institutions (WG 3). WG 4 aims to create a framework for training methods. Finally, WG 5 is responsible for communication, dissemination, and impact maximization of the activities of the other WGs. Additional leadership positions include the gerodontology coordinator, the qualitative research methodology advisor, the accreditation procedure advisor, and the translation coordinator.

As part of its activities, WG 1 developed an open online survey to map the educational interests and needs of health care professionals and final-year medical students across PROGRAMMING countries. This survey aims to describe the “current baseline” on which the WGs will construct the educational content in geriatric medicine and the framework of training methods for its delivery. This survey serves as a starting point to reach an evidence-based agreement on shared core competencies in the care of older adults in Europe. It will inform a competence-based core curriculum, which will be launched together with recommendations for educational frameworks in the future. In this paper, we describe the aim, development, structure, content, and dissemination of the survey. The complete survey is presented in Multimedia Appendix 1.

We hypothesized that health care professionals and final-year medical students will report (1) poor self-perceived knowledge, high relevance for clinical practice, and high interest in receiving further education and training in emerging geriatric topics such as delirium and deprescribing; and (2) higher self-perceived knowledge on most geriatric topics in case they had formal undergraduate education in geriatric medicine and clinical rotations in geriatric settings (ie, geriatric medicine acute care hospital wards and outpatient clinics, rehabilitation settings, and nursing homes) compared to not having had it. If the survey’s results confirm these hypotheses, we will advocate for the inclusion of emerging geriatric topics in professional educational curricula and for clinical rotations in geriatric settings. Furthermore, we hypothesized that (1) the educational interests and needs, (2) the barriers to receiving education and training in geriatric medicine, and (3) the preferred mode of delivery (ie, in person, online, or hybrid) of courses and the teaching methods will vary by country of education, country of current work, and setting of work. In addition, we hypothesized that different clusters of countries may emerge with respect to the educational needs of health care professionals in geriatric medicine based on previous undergraduate and postgraduate geriatric education and different health care system organizations. By obtaining insights into this diversity, we will be able to tailor our educational efforts to the needs of specific countries and settings of work.

### Objectives

This paper outlines a mixed quantitative and qualitative approach that was used to evaluate educational needs in geriatric medicine. This methodology could also be of value in other specialty areas that are seeking to improve understanding of knowledge gaps and needs for health care professionals and medical students.

## Methods

### Development of the Survey

**Figure 2:**
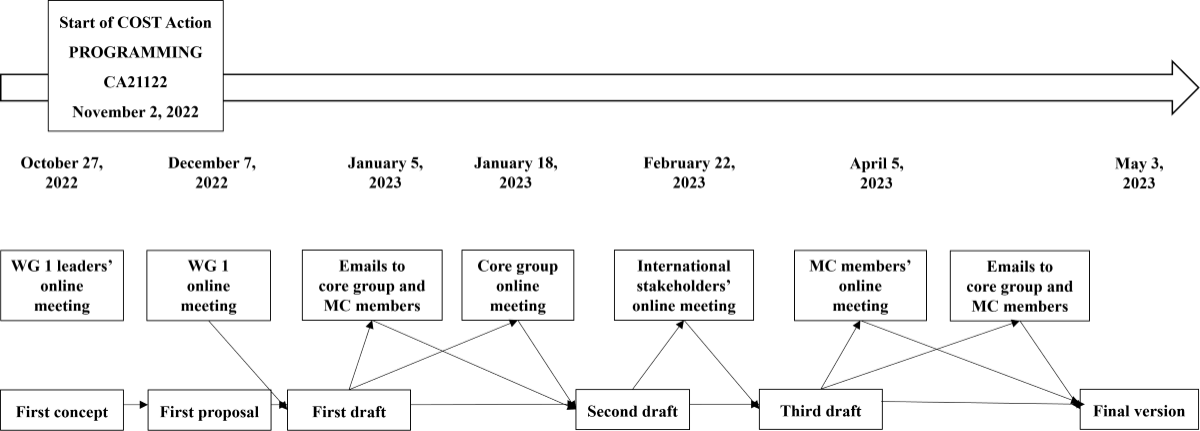
Development of the PROmoting GeRiAtric Medicine in countries where it is still eMergING (PROGRAMMING) survey. COST: European Cooperation in Science and Technology; MC: managing committee; WG: working group.

The survey was created by members of WG 1 and the core group, who are experts in the care of older adults from a variety of health care and non–health care professional backgrounds and from several European and neighboring countries. It was developed through a cocreation process of discussions and feedback that started in October 2022 and was completed in May 2023 (Figure 2).

In October 2022, just before the start of PROGRAMMING, a few WG 1 members conceived the first survey proposal. They discussed this in the first online meeting of WG 1 members in December 2022. In January 2023, they created and emailed a first draft to the core group and MC members. The core group provided feedback during an online meeting in January 2023, and a second draft was created. In parallel, the MC members were asked to identify stakeholders—health care professionals or policy makers with interest and expertise in the care of older adults—in PROGRAMMING-involved countries. They created a panel of 68 international stakeholders from 24 countries (Multimedia Appendix 2) who gave feedback on the survey during an ad hoc online meeting in February 2023. After the meeting, individual stakeholders emailed further feedback to WG 1 members. On the basis of this, a third draft was developed and presented to the MC members in an online meeting in April 2023, and strategies for dissemination of the survey were also discussed. Following further revision and testing of the online version, the final version of the survey was created in English in May 2023 (Multimedia Appendix 1).

WG 1 did not apply a rigorous Delphi process to the development of the survey; for instance, the feedback was not anonymous. However, in line with the Delphi process, WG 1 assumed group judgments to be more valid than individual judgments [29,30]. On the basis of feedback from the international stakeholders, WG 1 adopted the phrase “care for older people” instead of “geriatric medicine” in the survey as the former was deemed more inclusive of health care professionals other than physicians or surgeons. On the basis of the feedback, WG 1 decided to delete, add, or modify specific questions, response options, or entire sections of the survey. For instance, the management of heart failure was initially included and then excluded from the list of topics and skills relevant to the care of older adults. A question on extracurricular volunteer work with older adults was added as this may promote more positive attitudes of medical students toward older adults [31,32].

### Content of the Survey

#### Overview

**Figure 3:**
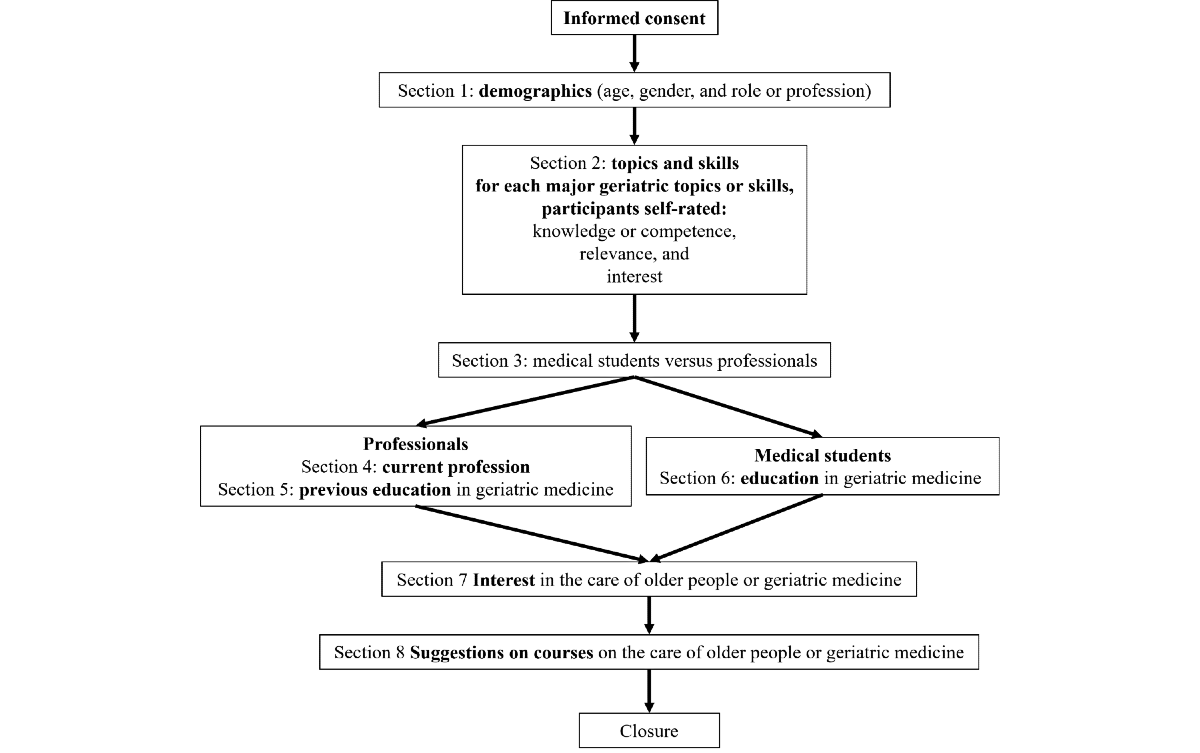
Structure of the PROmoting GeRiAtric Medicine in countries where it is still eMergING survey.

The survey targeted a broad spectrum of health care and non–health care professionals and final-year medical students, as detailed in the *Informed Consent* section (Multimedia Appendix 3). The survey sections are described in the following manuscript sections and summarized in Multimedia Appendix 3 and Figure 3.

#### Informed Consent

This section informed the potential participants of the aim of the survey and that it was part of the PROGRAMMING CA21122 funded by the EU. It also stated that the EuGMS is the grant holder of PROGRAMMING. The “Disclaimer” informed the potential participants of the EuGMS privacy policy in compliance with the General Data Protection Regulation (GDPR; EU 2016/679) and of the expected duration of the survey (10-15 minutes). It informed them that they could provide their email address in case they wished to receive information or educational material relevant to PROGRAMMING and the EuGMS. To comply with the national ethics regulation, the German version of the survey did not include the option to provide an email address.

#### Section 1: Demographics

This section included 3 questions on gender (man, woman, and “prefer not to say”), age (16 years to ≥100 years or “prefer not to say”), and role or profession. For role or profession, the participants could only choose one of the options in the list or the option “other” and describe in free text their current role or profession. For example, retired or unemployed professionals and professionals with more than one occupation could choose “other.” The question on gender was included to explore gender differences in attitudes toward caring for older adults.

#### Section 2: Topics and Skills

The longest section of the survey started with an explanation of its aim and then included 33 questions related to major geriatric topics and skills, as detailed in Multimedia Appendix 1. For each topic or skill, the participants rated their knowledge or competence on a 5-point Likert scale as very low, low, fair, high, or very high. Then, using the same scale, they rated the relevance of this topic or skill to their current or future work or clinical practice and then their interest in receiving further education or training on each topic or skill. The final question asked the participants to rate their “overall knowledge and competence in care of older people” in relation to what was needed for their role or profession.

#### Section 3: Medical Students Versus Professionals

This section was meant to divide professionals and final-year medical students. Professionals were directed to sections 4 and 5 and then sections 7 and 8. Final-year medical students were directed to sections 6, 7, and 8.

#### Section 4: Current Profession (Professionals)

This section inquired about the main qualification or degree, the year when it was obtained, and the country or main country of qualification. These questions considered the developments in teaching and training that took place in the relevant country over the years and the heterogeneity of teaching and training between countries.

Further questions asked whether medical doctors had a medical specialty and, if so, the country and the year in which this was obtained. Another question asked about additional specialties or subspecialties. Question 4.8 asked health care professionals other than physicians or surgeons whether they had a specialty or subspecialty or competency in the care of older adults. Question 4.9 asked all health care professionals in which country they currently worked. Questions 4.10 to 4.13 explored the settings of current work. Question 4.14 asked about the years of experience caring for older adults. Question 4.15 asked about the proportion of older adults among the patients of the clinical health care professionals.

#### Section 5: Previous Education in Geriatric Medicine (Professionals)

It was important to explore whether health care professionals had previous formal training in the care of older adults for many reasons. First, exposure to geriatric medicine and role models in this specialty may influence health care professionals to choose the specialty or subspeciality of geriatric medicine in countries where it exists [33]. It may also reflect their self-perceived knowledge on major geriatric medicine topics. Therefore, this section asked the health care professionals whether they attended specific courses or lectures on the care of older adults or geriatric medicine.

Then, it inquired about clinical rotations in each of the following settings: a geriatric medicine acute care hospital ward, a rehabilitation setting, and a care home or nursing home or a geriatric medicine outpatient clinic. Care homes or nursing homes have recently been advocated as training settings, although this is uncommon in many countries [34]. Finally, this section asked about volunteer work with older adults and research in the field of geriatric medicine.

#### Section 6: Education in Geriatric Medicine (Medical Students)

The first 4 questions in this section targeting medical students were intentionally similar to those in section 5, which targeted professionals. Medical students were asked about specific courses or lectures, clinical rotations, volunteer work, and research related to geriatric medicine. Questions 6.5 and 6.6 asked them about the country (or main country) and university where they were studying. Question 6.7 asked them whether they wanted to become geriatricians. Question 6.8 explored the potential obstacles to becoming a geriatrician. In question 6.9, medical students were asked about their “thoughts on becoming a geriatrician” and given the option of writing free text. Finally, they rated how prestigious it is to be a geriatrician in their country or selected that “there are no geriatricians” in their country.

#### Section 7: Interest in the Care of Older People or Geriatric Medicine

In this section, both professionals and medical students rated their “satisfaction and feeling comfortable when interacting with older adults in both professional and non-professional settings.” Health care professionals also rated these feelings specifically “in a professional setting.” Both professionals and medical students were then asked about their “thoughts on caring for older people” and given the option of writing free text. Finally, they rated how prestigious it is to care for older people in their country.

#### Section 8: Suggestions on Courses on the Care of Older People or Geriatric Medicine

This section asked professionals and medical students about the acceptability and perceived effectiveness of a variety of educational and training methods. It asked them to rate their interest in attending courses on the care of older adults and whether they would prefer in-person, online, or hybrid courses. It explored the barriers to attending these courses. Finally, it asked the respondents to rate the effectiveness of various teaching methods, including A to Z textbook such as lectures on a topic, teaching of clinical tools for screening or diagnosis and evaluation, question-driven teaching, clinical cases, role-plays, short presentations by the participants as a learning tool, and assignment of group activities. Question 8.6 gave them the option to suggest any other teaching methods.

#### Final Section

The last section gave the participants the option to provide a contact email address. This option was deleted in the German version of the survey.

### Translations of the Survey

The survey was originally developed in English and then translated into Albanian, Bosnian, Bulgarian, Croatian, Czech, Danish, Dutch, French, German, Greek, Hebrew, Hungarian, Italian, Latvian, Lithuanian, Macedonian, Polish, European Portuguese, Romanian, Serbian, Slovak, Spanish, Swedish, and Turkish. The translations aimed to promote participation among those who did not have English as their first language and adopt an inclusive attitude toward diversity within health care.

The translations followed specific guidelines by WG 1 (Multimedia Appendix 4). The survey was directly translated only from English into each of the other languages. Each translation required at least a translator and a proofreader. In most cases, the translators were PROGRAMMING members with a health care background who translated from English into their own native language. All proofreaders were PROGRAMMING members and mostly MC members. For each translation, a form was created and tested by providing mock responses that were checked for alignment with the English-language version. Random errors were corrected, and mock responses were deleted before dissemination.

### Identification of Stakeholders

**Figure 4:**
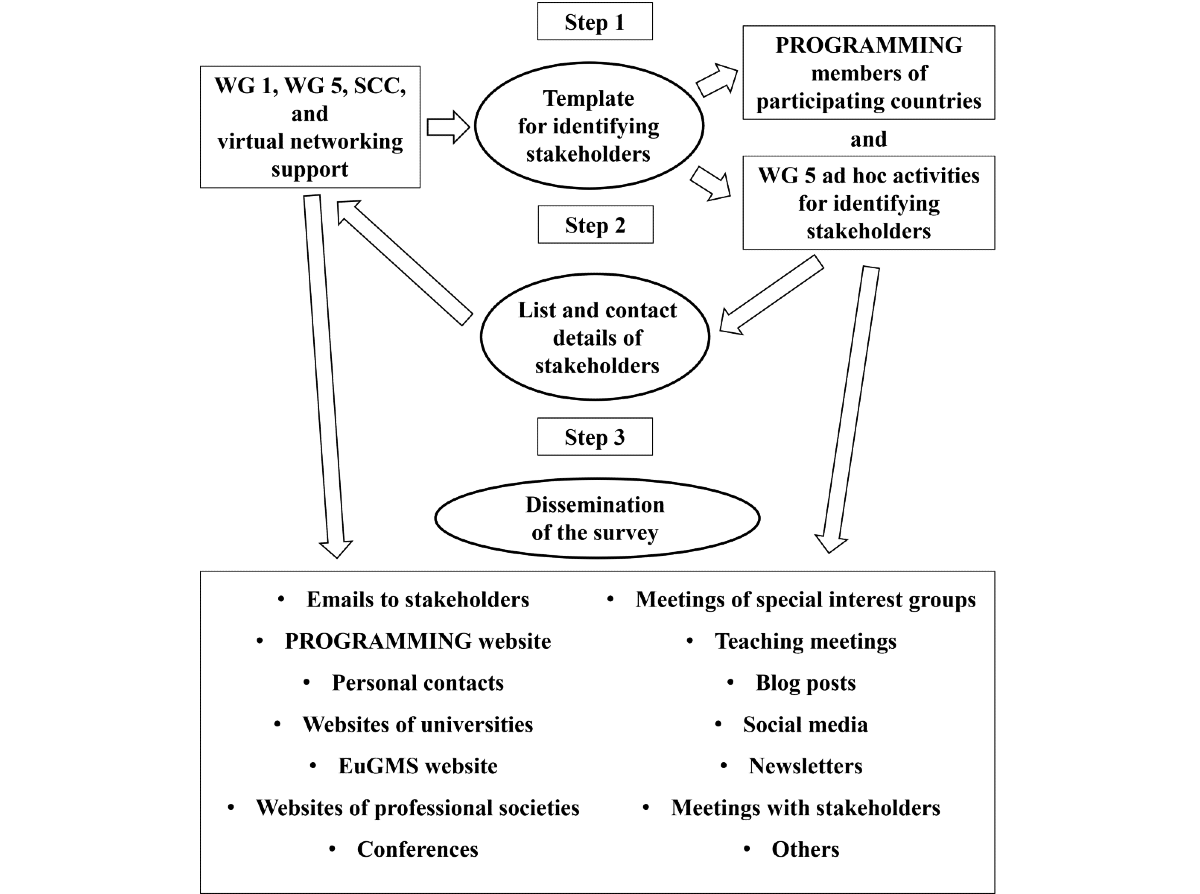
Dissemination of the PROmoting GeRiAtric Medicine in countries where it is still eMergING (PROGRAMMING) survey. EuGMS: European Geriatric Medicine Society; SCC: science communication coordinator; WG: working group.

WG 1 and WG 5 aimed to identify relevant stakeholders who could promote the dissemination of the survey to specific countries or internationally (Figure 4). WG 5 developed a framework to identify and categorize potential stakeholders and a guide to facilitate discussion group meetings held for identifying the stakeholders internationally and at the country level [35].

The MC members or, in countries with no MC members, PROGRAMMING members were asked to identify relevant stakeholders from their country and create lists of these using an official template [35]. On this template, the stakeholders were classified as those related to education (including undergraduate and postgraduate education and continuous professional development), policy, nongovernmental nonprofit societies, scientific societies, educational societies, professional societies, student organizations, charities, and research. The same stakeholder could be named in more than one section, and their publicly available contact details were included. WG 1 and WG 5 asked the stakeholders to forward the survey to the members of their organizations without obtaining direct access to their contact lists.

WG 1 and WG 5 encouraged the adoption of this template, although it was not mandatory, and the creation and completion of a list of stakeholders per country before October 2023. At the same time, until April 2024, WG 1 and WG 5 accepted new lists as well as any later corrections or additions. The number of stakeholders varied between a few (<10) and a few hundred per country. WG 1 and WG 5 encouraged the identification of international stakeholders such as European professional or scientific societies.

### Strategies of Dissemination

**Figure 5:**
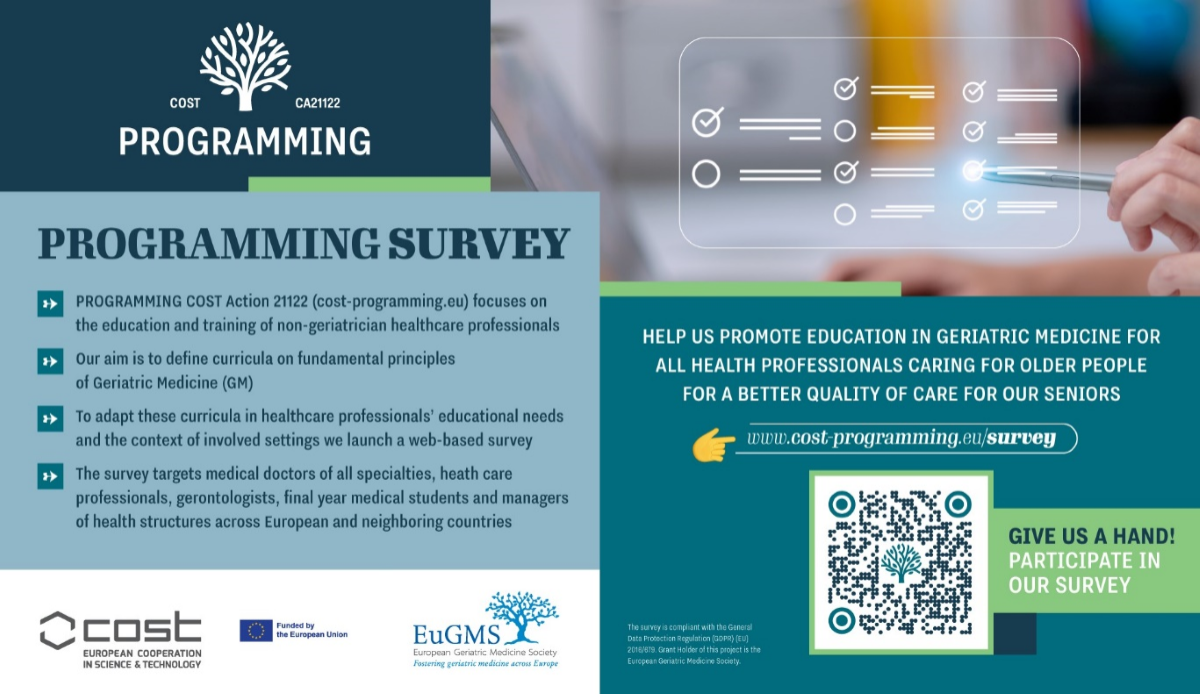
Official poster for the dissemination of the PROmoting GeRiAtric Medicine in countries where it is still eMergING (PROGRAMMING) survey.

The survey was disseminated exclusively online and through several strategies (Figure 4). A specific page was created and dedicated to the survey on the PROGRAMMING website, making the online survey forms openly accessible to the website visitors [36]. The Action awarded a Virtual Networking Support grant to the EuGMS secretariat for the online dissemination of the survey. The EuGMS secretariat contacted international and national stakeholders via emails that described the aim of the survey, the compliance with the GDPR, and the categories targeted by the survey. These emails included direct links to the survey in one or more chosen languages (Multimedia Appendix 5). They were written in English and then adapted and translated into national languages, as deemed appropriate for local contexts. Later, simplified versions were created, and the official poster for dissemination was attached to them (Multimedia Appendix 6 and Figure 5). In a few countries, such as Belgium, France, Portugal, and Finland, the MC members contacted the stakeholders directly without the help of the EuGMS secretariat.

**Figure 6:**
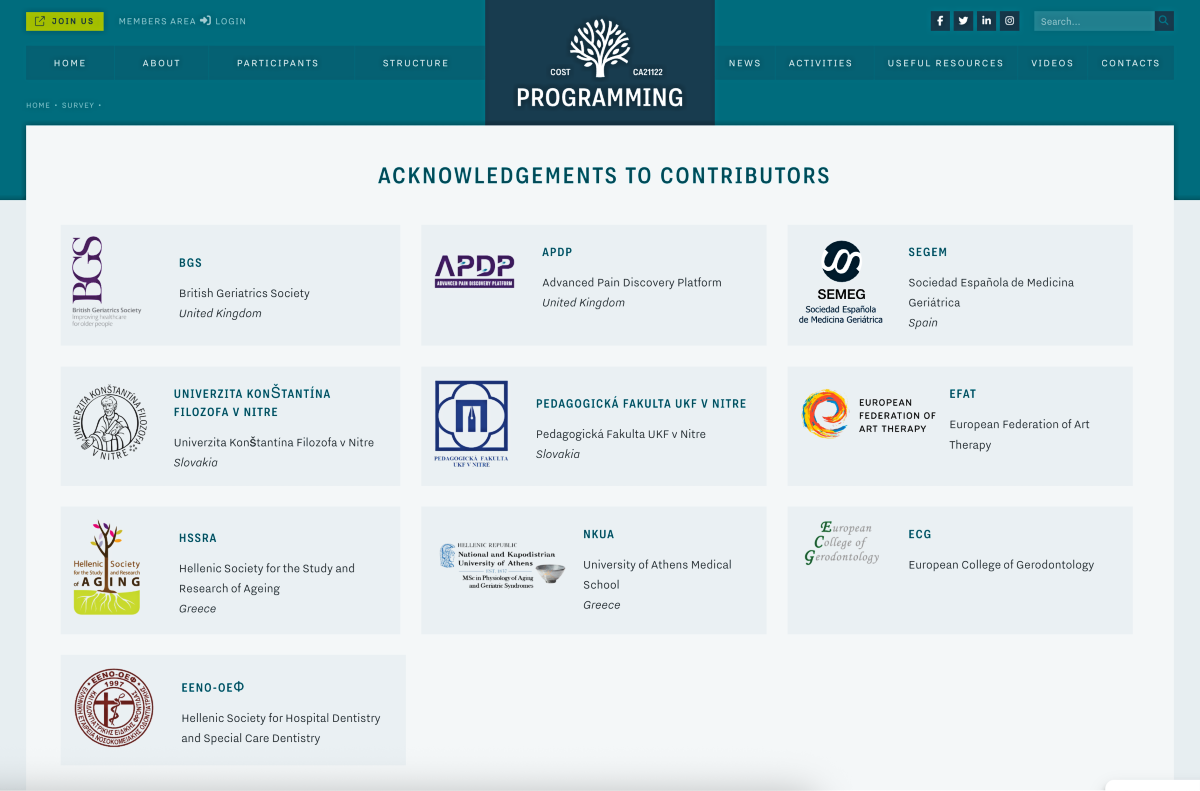
Academic institutions and professional societies that contributed to the dissemination of the survey.

In parallel, in all countries, WG 1 and WG 5 encouraged dissemination through social media and professional contacts (Figure 4). In December 2023, WG 1 and WG 5 held a webinar on the potential strategies for dissemination of the survey with approximately 30 PROGRAMMING members. A few blog posts were posted on the websites of professional scientific societies. We publicly thanked the academic institutions and professional societies that contributed to the dissemination of the survey (Figure 6).

### Ethical Considerations

Ethics approval was obtained in the countries where local regulations necessitated review by an ethics committee. The approval numbers and specific institutional review boards overseeing the study are documented in Multimedia Appendix 7. In countries where ethical review was not required, local regulations and institutional policies were followed.

The survey protocol received ethics approval from the Jagiellonian University ethics committee, Kraków, Poland (118.6120.61.2023; request submitted on May 26, 2023; approval by the ethics committee on June 5, 2023); the Ethics Committee for Human Research, Faculty of Medicine, Ss. Cyril and Methodius University in Skopje, Republic of North Macedonia (03-338211; dated August 7, 2023); Medical University of Graz, Austria (35-373 ex 22/23; voted and approved on September 15, 2023); the Faculty of Medicine, Health, and Life Sciences Research Ethics Committee of Queen’s University Belfast, United Kingdom (23_123; September 27, 2023); Jerusalem College of Technology, Jerusalem, Israel (014_23; September 2023); the ethics committee of the Faculty of Dental Medicine, University of Belgrade, Serbia (36/42; dated October 16, 2023); the Committee of Bioethics and Deontology, School of Medicine, National and Kapodistrian University of Athens, Athens, Greece (762; October 23, 2023); Istanbul Faculty of Medicine Clinical Research Ethics Committee, Istanbul, Türkiye (application 2023/2339; decision 26; dated December 29, 2023); and the Ethics Commission of the Faculty of Medicine of the University of Cologne, Germany (application 23-1439; letter from the Ethics Commission dated March 11, 2024). The Research Ethics Committee of “Carol Davila” University of Medicine and Pharmacy, Bucharest, Romania (12996; dated May 10, 2023); the Central Denmark Region Committees on Health Research Ethics, Denmark (request 173/2023; outcome letter dated November 10, 2023); the secretariat of the Umbria Regional Ethics Committee, Italy (email dated September 13, 2023); and the Research Ethics Committee of Rīga Stradiņš University, Latvia, stated that the study may be conducted without an approval from the committees. The ethics committee of Constantine the Philosopher University in Nitra, Slovakia, approved the project implementation and questionnaires, including documentation (UKF/370/2025/191013:006). The Ethics Committee of General Hospital “Prim. Dr. Abdulah Nakas,” Sarajevo, Bosnia and Herzegovina, approved the project implementation and questionnaires, including documentation (26-213-139/25; Sarajevo, March 3, 2025). According to the Republic of Lithuania’s Law on Ethics of Biomedical Research (May 11, 2000; VIII-1679) and the assessment of the Lithuanian Bioethics Committee, the permission of the Lithuanian Bioethics Committee was not required for this survey targeting professionals; medical students were not targeted in Lithuania. According to the Swedish Ethical Review Authority, the Act on Ethical Review of Research Involving Humans (2003:460) states that “If you have designed a study in such a way that sensitive personal data will not be collected ethical review is not required.” In Albania, ethics approval was not required for this survey with deidentified data (law 80/2015, “On Higher Education and Scientific Research in Higher Education Institutions of the Republic of Albania”). In Belgium, Bulgaria, Croatia, the Czech Republic, France, the Netherlands, Portugal, and Spain, ethics approval was waived as the survey collected data that were deidentified, there were no interventions, informed consent was obtained by proceeding with the survey, and participants were informed of the use of their data in accordance with the GDPR (EU 2016/679), ensuring compliance with the applicable regulations.

Participants were required to provide informed consent before completing the survey. The first section of the survey, “Informed consent,” included the following: “Disclaimer If you continue, you are giving your informed consent to this survey and you are accepting the EuGMS privacy policy. The EuGMS is compliant with the GDPR (EU) 2016/679.” It provided a link to the EuGMS website for further information on “how the EuGMS collects, keeps, and processes private information in compliance with GDPR” and the contact email of the EuGMS secretary. The participants could opt out of the survey while filling it out, and their responses were not recorded. In addition, they may contact the EuGMS secretary to opt out of the survey as specified on the survey web page of the PROGRAMMING website [26]. As of February 2025, no participant has contacted the EuGMS secretary asking for their data to be deleted.

All survey responses were anonymized to prevent identification of individual participants. Data were stored securely on password-protected servers, with access restricted to authorized researchers. No personally identifiable information was collected, ensuring compliance with data protection regulations such as the GDPR.

No monetary or material compensation was provided to participants. Participation was entirely voluntary and based on professional and academic interest. We believe that the benefit to participants will be indirect and in the long term as we hope that our research will promote geriatric medicine across Europe and beyond. We publicly thanked the participants on the PROGRAMMING website [26].

### Quantitative Analyses

**Figure 7:**
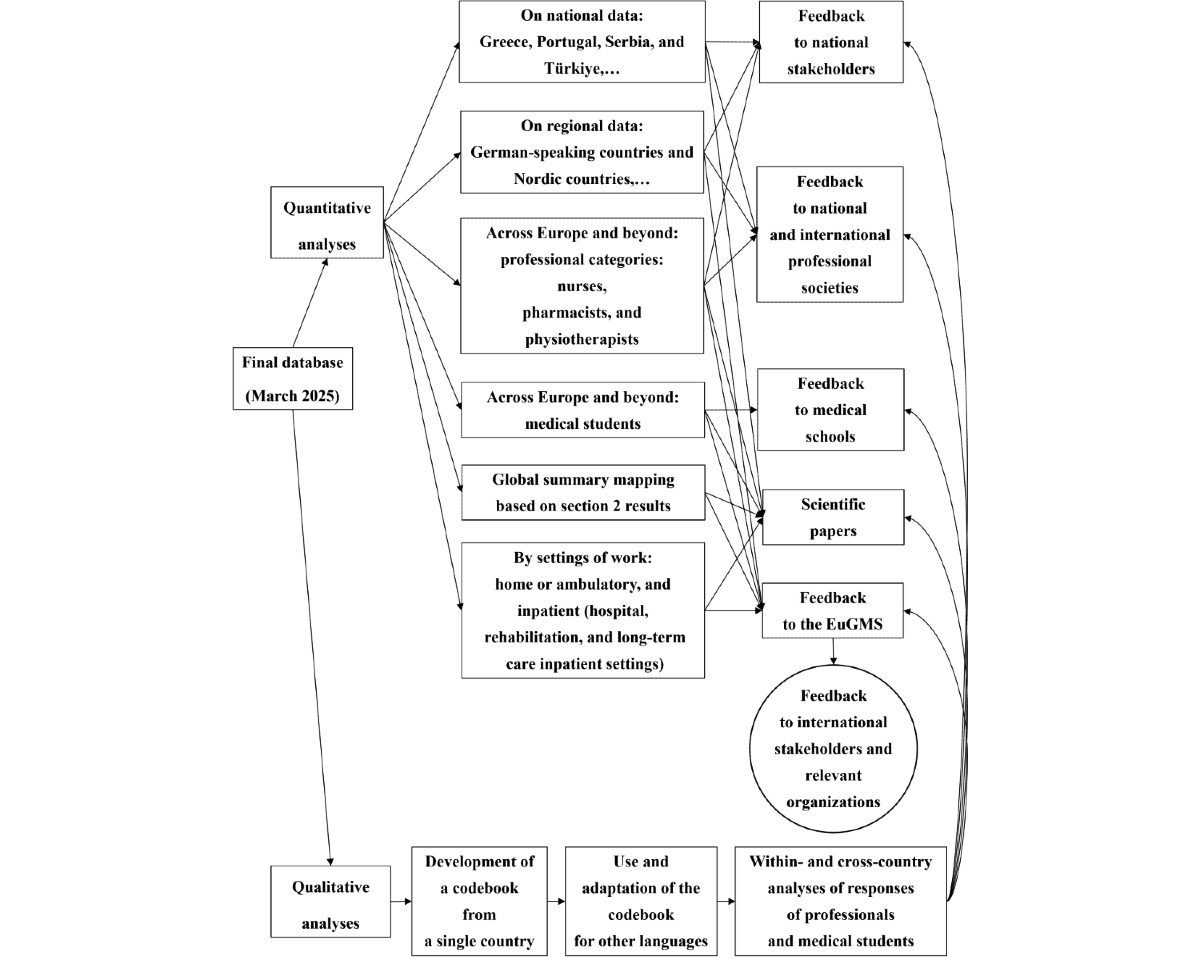
Analyses of the survey data. EuGMS: European Geriatric Medicine Society.

We are planning quantitative and qualitative analyses in parallel (Figure 7). Various national and cross-country teams have volunteered to conduct quantitative analyses of the survey data in parallel following approval of their research proposals. There are national teams that will analyze the data of their own countries and share them with national stakeholders and professional societies (eg, in Greece, Portugal, Serbia, and Türkiye), as well as broader regional teams (eg, German-speaking countries and Nordic countries). Teams with members from at least 3 different countries will run analyses of data from across Europe and beyond, focusing on the responses from 1 specific professional category (eg, nurses, pharmacists, and physiotherapists) or from medical students. WG 1 members will conduct global summary analyses of the section 2 findings to create “benchmarking” and mapping data. Finally, WG 2 will explore the responses of professionals working in ambulatory settings as defined on the PROGRAMMING website [26]. In parallel, WG 3 will analyze the responses of professionals working in inpatient settings, including acute care general and psychiatric hospitals, rehabilitation hospitals, long-term care psychiatric hospitals, and long-term care institutions such as residential and nursing homes [26].

The statistical analyses will include basic descriptive statistics such as frequencies (and percentages) for categorical variables (eg, role or profession and self-perceived knowledge on each geriatric topic) as well as means with SDs and medians with ranges and IQRs for continuous numerical variables such as age. We will test for differences in self-perceived knowledge on each geriatric topic between different categories of health care professionals (eg, nurses vs all other professionals) by using chi-square tests, as well as for perceived relevance to clinical work and interest in receiving further education on each geriatric topic. In view of cross-country differences in health care and educational systems, we will conduct sensitivity analyses within countries (at least in those with enough responses). Furthermore, we will explore differences in knowledge on each geriatric topic and overall across countries and settings of work using chi-square tests.

We will conduct analyses within the same professional category (eg, nurses) to test for the role of previous formal education in geriatric medicine (or rotations in geriatric clinical settings) in promoting knowledge on each geriatric topic. Previous formal education in geriatric medicine will be the determinant. Self-reported knowledge on a specific geriatric topic (eg, delirium) will be the dichotomous outcome (poor vs good). In our survey, self-reported knowledge on each geriatric topic was measured using a 5-point Likert scale (from “1=very low” to “5=very high”). In the chi-square analyses, we will show the full descriptive statistics of our data and the 5 categories of self-reported knowledge. For summary purposes, we will then dichotomize self-reported knowledge as either poor (very low or low knowledge) or good (fair, good, or very good knowledge).

We will conduct binary logistic regression analyses with previous formal education in geriatric medicine as the determinant and self-reported knowledge of delirium as the outcome only in the sample of a single professional category; these analyses will be adjusted for age, gender, and country. We expect that those with previous formal education in geriatric medicine will report good knowledge of delirium more frequently than those without it. We will repeat these analyses for each of the geriatric topics of section 2. Similarly, we will conduct binary logistic regression analyses with previous rotations in geriatric settings (acute care geriatric ward, outpatient clinic, rehabilitation ward, and nursing home) as the determinant and knowledge on each geriatric topic as the outcome. We expect that, within each professional category, those with previous rotations in geriatric settings will report good knowledge on geriatric topics more frequently than those without them. Binary logistic regression is a statistical analysis that is suitable for binary outcomes such as poor versus good self-reported knowledge.

We will organize meetings between WG 1 experts in quantitative and qualitative analyses and national and cross-country researchers to ensure consistent data analysis protocols. At the same time, we will welcome adaptations of the analyses to national needs. We are not planning any weighted analyses; we welcome sensitivity analyses and particularly within-country analyses in view of the disproportionate number of responses in a few countries.

### Qualitative Analyses

We are planning qualitative content analyses of the free-text responses to the questions exploring professionals’ and medical students’ thoughts on caring for older people and medical students’ thoughts on becoming geriatricians. Undertaking qualitative analysis for such linguistically diverse data presents challenges and dilemmas [37]. The responses are in >20 languages. Some concepts may not be easily translatable, and capturing nuances of terminology between languages, perhaps reflecting cultural and professional particularities, may generate significant time and resource demands. The responses may also be interpreted differently depending on the professional background of the researchers.

There is a further tension between having a standardized approach but also one that is inductive and can cope with the full richness and context of the sentiments expressed. Across Europe and beyond, broad emotions and sentiments have emerged from the responses, ranging from respect and admiration to sarcasm and feeling that older people are discriminated against by society or family members. Caring for older people is described as both rewarding and challenging. Words such as “enriching” and “gratitude” are mentioned, but also “ageism,” “discrimination,” and “cuts.”

To overcome these multi-language challenges, we developed the following analysis process based on content analysis. Content analysis aims to identify and interpret meaning within recorded communication through labeling smaller data pieces and organizing them in a way that describes or explains a phenomenon [38]. It was originally used to quantitatively describe the manifest content of communication but was adapted to include more qualitative approaches to analysis to understand deeper meanings [39]. In total, 2 coders fluent in the response language will independently code the responses using Microsoft Excel. Coding will be conducted in the coders’ native language; bilingual and culturally knowledgeable researchers will ensure both linguistic accuracy and cultural nuances. A standard coding framework will be developed and iteratively refined through cross-cultural discussions to maintain comparability across languages. Discrepancies will be resolved through consensus meetings incorporating responses from native speakers and subject matter experts. To mitigate linguistic and cultural biases, coding checks and double validation will be conducted across diverse linguistic contexts.

The focus will be on manifest content, but latent content will also be coded as responses tend to be fairly brief but can contain emotive words open to interpretation (eg, “must”). To ensure that multiple possible interpretations of any latent sentiments are explored and consistently applied, 2 coders will be involved and will discuss any disagreements by consulting qualitative experts and the wider WG 1 as needed. This will also help address the issue that the coding researchers may bring biases associated with their own professional background and understanding, and they will be encouraged to be reflexive regarding this throughout coding and discussion.

Multiple codes will be applied where needed; for example, “Perkujdesje, respekt, dashuri, mirenjohje, dhembshuri” (Albanian), which translates to “Care, respect, love, gratitude, compassion,” will generate 5 codes. The codebook will be agreed upon between 2 researchers, and logical category groupings will be applied. Bearing in mind the limited data per person, categories rather than themes will form the main basis of analysis [40], focusing on describing similarities and differences on a manifest and descriptive level related closely to the original responses but including latent data where relevant. Frequencies will be applied to demonstrate the prevalence of views within these categories, especially where there are larger numbers of free-text responses, in addition to textual description. The process will be initially piloted on the Albanian-language responses and will be refined as needed.

The Albanian-language coding process will be conducted in the Albanian (native) language to preserve linguistic and cultural nuances. In total, 2 independent researchers will code the data separately, ensuring reliability, under the guidance of a qualitative research expert. It is planned that teams working in other languages will be able to use the Albanian-language codebook, which will be translated into English, as a basis for coding. Any language- or culture-specific concepts will be described as clearly as possible in English, with the original-language words retained in brackets for clarity. The Albanian-language codebook will serve as a basic framework and applied deductively where relevant codes already exist to maximize comparability between languages for larger-level analysis but taking an inductive approach to ensure that any new data are captured. Inductive coding will be applied for emerging or unique data that do not fit into existing categories. New codes will be discussed in consensus meetings and incorporated into the codebook through an iterative refinement process.

New codes will be added to the codebook iteratively for each country’s analysis following the same principles mentioned previously. Other countries can choose to use the original or adapted codebooks and will be encouraged to discuss new codes with the Albanian-language team and the wider WG 1, particularly if they are unclear on the meaning and comparability of certain codes or definitions. WG 1 will store a copy of all codebooks and will review and refine the initial codebook with the Albanian-language team. Quotes will be translated into English for publication.

## Results

### Timeline

The dissemination of the survey started on October 9, 2023. Although the initial aim was to have the same time window for dissemination for all languages and countries, this proved to be not possible. The ethics applications in a few countries required long periods, and other countries joined this initiative after the start of dissemination. Dissemination ended on April 30, 2024, for most countries, whereas it was prolonged until June 5, 2024, for the German and Hungarian surveys.

### Social Media Metrics

The survey was posted on the PROGRAMMING website and on the social media of the EuGMS and other professional societies. In 2023, the PROGRAMMING website [26] had 30,252 visits from <15,674 unique visitors; in 2024, it had 94,784 visits from <38,019 unique visitors. In addition, Multimedia Appendix 8 presents some metrics that are an underestimation of the overall dissemination.

### Final Database

As of February 2025, WG 1 is merging and coding the survey data from multiple electronic forms into a unique database using SPSS (version 29; IBM Corp) [41]. This database will include all the responses that were collected by the survey; it will retain the open-ended responses in the original languages and then add their translations. From June 2024 to January 2025, WG 1 conducted preliminary analyses and developed examples of visual presentation of preliminary data.

As of April 2025, WG 1 has finalized the cleaning of the database. The survey received 6099 responses; as of April 2025, the final cleaning has led to 5922 responses, including 5474 (92.43%) from health care professionals and 448 (7.57%) from medical students. Preliminarily, it included responses from dieticians (3%), dentists (8%), medical doctors in training or not in training (37%), medical students (7.5%), nurses (14%), pharmacists (6%), physiotherapists (6%), and other categories. Responses were excluded from the final database in case the respondent was not a professional or a medical student (eg, responses from dental or nursing students or students other than medical students were excluded), the respondent was a professional who erroneously filled in the student section 6 rather than sections 4 and 5 for professionals, the respondent was a medical student who erroneously filled in the professional sections 4 and 5 rather than the student section 6, there were multiple entries from the same individual, or there were serious inconsistencies across the responses from the same individual.

WG 1 researchers carried out log file analyses to prevent multiple entries from the same individual by checking for duplicates in the optional email addresses provided and by comparing the responses of participants with exactly the same demographics using the tool for identifying duplicate cases in SPSS. When duplicates were found, only their first entry was retained in the final database, whereas the most recent one was excluded, except in the case of a medical student and cases in which the responses in the likely native language were kept instead of those in English.

WG 1 researchers also checked the internal consistency of the responses by creating cross-tables for variables such as profession (question 1.3: “Role or profession”) and main qualification (question 4.1: “Main qualification or degree”) and by checking the age of the respondent against the year of main qualification or degree and the year of medical specialty (in the case of medical specialists). WG 1 sought clarification from national PROGRAMMING members. In case of serious and multiple inconsistencies, the entire set of responses was excluded from the final database. In case of isolated unrealistic entries (eg, the year of medical specialty was the expected one), this was highlighted as unrealistic with a note within the coded or translated response. WG 1 researchers conducted an extensive cross-checking of the consistency of the responses on the settings of work of professionals (questions 4.10 to 4.13).

There were practically no incomplete questionnaires. All questions in the survey were intentionally made mandatory to avoid incomplete or partially completed questionnaires. Even questions with free-text responses were made mandatory, and the respondent was invited to write “blank” or “not applicable” as appropriate.

We cannot measure the time that each respondent needed to fill out the questionnaire; the variable “time stamp” refers to the date and time of submission of the questionnaire. We cannot exclude questionnaires that were submitted too soon, but no respondent could submit the questionnaire unless all mandatory questions were answered. It is likely that many potential respondents started to fill out the questionnaire but then abandoned it; their responses were not recorded. We cannot estimate the completion rate of the survey.

No response was deleted. The responses that were excluded from the final database were deidentified for confidentiality and retained in separate files for documentation.

## Discussion

### Expected Findings

We developed a web-based survey to map the educational interests and needs of health care professionals and final-year medical students across the countries of the COST Action PROGRAMMING CA21122. This paper describes the development, structure, and dissemination of the survey in line with current guidelines [42]. We have attached the original English-language version of the survey (with a few technical instructions) to facilitate future replication or adaptation of our work (Multimedia Appendix 1).

Through the implementation of our survey, we collected responses on the educational needs in geriatric medicine from 5474 health care professionals and 448 medical students across Europe and beyond. We achieved this by organizing a dissemination strategy that involved recognized leaders in health science education, following appropriate ethics regulations, and offering translated versions of the survey into several European languages to overcome language barriers that could limit self-expression of needs. In addition, we managed to have significant representation from many categories of health care professionals comprising the geriatric interdisciplinary team across the PROGRAMMING countries (ie, the health care professionals from different disciplines who collaborate to provide comprehensive and coordinated care to older adults). This will allow us to adapt our suggested geriatric educational curriculums to the different needs of health care professionals and across the widely diverse health care landscape across Europe and beyond.

Our survey adds to previous literature exploring the attitudes toward caring for older people and choosing career pathways in this field [43-48]. For example, previous surveys have explored the interest in geriatric psychiatry of Canadian psychiatry residents [43]; the knowledge on and attitudes toward geriatrics of medical students, internal medicine residents, and geriatric medicine fellows at an American academic medical center [44]; and the attitudes toward older adults of medical students at a US medical school [45], clinical pharmacists from a Canadian province [46], dentists from a Brazilian city [47], or physiotherapists [48]. The novelty of our survey is the targeting of a broad range of health care professionals, including those who will not specifically work in geriatric medicine but will encounter older adults in their clinical practice. We acknowledged that most older adults will receive care from medical doctors who are not geriatricians, as well as other members of multiprofessional teams [49]. While previous surveys have focused on a single health care professional category, a single country or academic center, and limited geriatric topics, our survey targeted multiple professional categories across Europe and beyond and explored a wide range of topics. Furthermore, we surveyed professionals and medical students in countries where geriatric medicine is still emerging and included this as a barrier. Similarly to most previous surveys, our survey was cross-sectional and could not capture any changes over time in medical students’ attitudes towards older people; in contrast, previous research conducted at a medical school in the United States described changes in medical students’ attitudes towards older people [45].

This study has several strengths. One of the major strengths is the broad geographic and professional representation, which enhances the relevance and generalizability of our findings. The use of a mixed methods approach also allows for a more comprehensive understanding of both quantitative trends and qualitative insights. A further strength was the harmonization and dissemination of the survey across several European and neighboring countries. One of the crucial points of discussion was whether the survey should be identical between nations or vary to reflect differences between states’ health care needs and systems. While many argued that the survey should be adapted to local contexts or tailored to respondents from different professions, others pointed out that comparisons can be made only when surveys are harmonized. In the end, the consensus was that the survey should be harmonized and include the same sections, questions, and response options across all countries. However, the way in which data are collected will allow for subgroup analyses for different countries and professions. While most sections were the same for both health care professionals and medical students, 2 sections specifically targeted health care professionals, and another one targeted medical students. Second, the survey was developed based on previous research on geriatric medicine education, including a recent literature review undertaken by PROGRAMMING members [16]. Third, the survey was developed through many rounds of discussion between professionals from different countries and professional backgrounds. Fourth, it was translated into >20 languages. In our view, this favored participation in the survey of potential respondents who are not fluent in English. In this way, our survey was more inclusive than surveys disseminated only in English or a specific national language. Fifth, it provided a detailed characterization of the educational background of the respondent. It collected data not only on the main qualifications of the respondents but also on the specific year and country of education. In this way, it identifies temporal trends in educational needs as well as cross-country variation. Sixth, it included questions on subjective or self-perceived knowledge on or competence in major geriatric topics alongside objective questions on courses and clinical rotations during formal education. In this way, it may detect gaps in self-perceived knowledge and explore their correlations with various types of training. Seventh, our survey targeted a broad range of health care professionals, which reflected the broad interdisciplinary and interprofessional approach required by contemporary geriatric medicine. Finally, our survey included open-ended questions (eg, on “thoughts about caring for older people”) with the possibility of free-text responses; a qualitative content analysis of these responses is planned, preferably in the original languages.

Some limitations should be acknowledged. First, the survey was not developed through a rigorous Delphi process, which may have influenced the selection of topics. Nevertheless, the iterative cocreation process with international stakeholders helped ensure relevance and comprehensiveness. Moreover, the survey was one exploratory step within the broader context of the PROGRAMMING initiatives, which included qualitative focus groups on areas not covered in the survey. A Delphi process would certainly be a necessary approach to final curricula and policy drafting. Second, selection bias may be present as participants with a strong interest in geriatrics may be more likely to complete the survey. To quantify this selection bias, we will calculate the proportion of medical students who would like to become geriatricians among our respondents and compare it with those reported in the literature. Previous studies in the United States have shown that approximately 4% of medical students would choose geriatric medicine as their medical specialty [50]. Third, the survey had a relatively long duration (10-15 minutes), which may have discouraged potential respondents and hindered a potentially wider dissemination. Fourth, the staggered dissemination of the survey may have limited participation in a few countries, but we do not expect this to affect the content of our findings as no major event with an impact on medical practice or education occurred in the few months of dissemination. Fifth, we disseminated our survey primarily in Europe. Consequently, the findings of our survey may not be generalizable to countries in other continents.

The findings of this study will inform the development of targeted educational curricula and professional training programs. We anticipate that insights from the survey will be shared with medical schools, professional societies, and policy makers to advocate for improved geriatric education. Future research should focus on validating these findings through objective assessments of knowledge acquisition and measuring the impact of educational interventions over time.

The goal of PROGRAMMING is to promote education on geriatric medicine to every health care professional caring for older adults in a way that is tailored to the professional roles, clinical settings, and country or region specificities.

At the same time, it is essential that the curricula of health care professionals be harmonized between countries for 2 main reasons [16]: first, to spread up-to-date scientific knowledge, evidence-based recommendations, and good clinical practice models so that older adults across Europe and beyond may receive appropriate care and, second, to remove barriers to the international mobility of health care professionals. Indeed, many high-income European countries are increasingly relying on the recruitment of health care professionals who were trained abroad within or outside Europe [10]. In 2019, one-third of the UK medical doctors were trained internationally [51,52]. The World Health Organization Global Code of Practice on the International Recruitment of Health Personnel (2010) recommended that all countries should train and retain sufficient physicians to meet demand rather than rely on immigration [10]. It supported circular migration of health care personnel so that skills and knowledge can be transferred to the benefit of both source and destination countries [10].

We plan to analyze the findings of the survey using both quantitative and qualitative techniques. We aim to identify the geriatric topics and skills in which health care professionals feel less competent, and we aim to explore patterns associated with lower self-perceived knowledge and competence, for example, in relation to country and qualification or professional role. Furthermore, we aim to explore gender differences in attitudes toward caring for older adults and, among medical students, in choosing geriatric medicine as a specialty. We also aim to investigate barriers, including gender-specific barriers, to choosing geriatric medicine as a specialty and attending courses on care for older adults. By conducting qualitative content analyses, we aim to obtain a deeper insight into the thoughts about caring for older adults of a broad number of health care professionals from diverse professional backgrounds in many countries in and beyond Europe.

One section of the survey explored the effectiveness of a broad range of teaching methods by asking health care professionals and medical students to rate them. Its findings may inform and guide the efforts of WG 4 of PROGRAMMING toward the implementation of education and training in the care for older adults in PROGRAMMING countries.

Furthermore, the survey collected detailed information on the settings in which health care professionals worked. This will allow for subgroup analyses on the educational needs of health care professionals by setting of clinical practice, including community settings, acute secondary care hospitals, and long-term care institutions. This will inform the activities of WG 2 and WG 3, which focus on developing the content of training of health care professionals.

### Conclusions

In conclusion, the findings of our survey will be used to inform educational projects across PROGRAMMING countries. We hope that similar surveys will be disseminated in countries that we did not cover, such as African, American, Asian, and Oceanic countries. We encourage policy makers and medical educators to use our survey or to draw inspiration from it to promote further development of geriatric medicine education (Multimedia Appendix 1).

## Appendix

Multimedia Appendix 1 The PROmoting GeRiAtric Medicine in countries where it is still eMergING (PROGRAMMING) survey on educational needs.

Multimedia Appendix 2 Composition of the international panel of stakeholders.

Multimedia Appendix 3 Sections of the PROmoting GeRiAtric Medicine in countries where it is still eMergING (PROGRAMMING) survey.

Multimedia Appendix 4 Translation guidelines.

Multimedia Appendix 5 Email to the stakeholders from Belgium.

Multimedia Appendix 6 Simplified email to the stakeholders from Norway.

Multimedia Appendix 7 Ethical considerations.

Multimedia Appendix 8 European Geriatric Medicine Society social media metrics.

Multimedia Appendix 9 Collaborators of the PROGRAMMING survey on educational needs.

Multimedia Appendix 10 Extended proposal form.

Multimedia Appendix 11 Simplified proposal form for analysis of the PROGRAMMING survey data on educational needs.

## Abbreviations

COST: European Cooperation in Science and Technology
EU: European Union
EuGMS: European Geriatric Medicine Society
GDPR: General Data Protection Regulation
MC: managing committee
PROGRAMMING: PROmoting GeRiAtric Medicine in countries where it is still eMergING
WG: working group

## Data Availability

The English-language version of the Promoting Geriatric Medicine in Countries Where It Is Still Emerging (PROGRAMMING) survey is available in Multimedia Appendix 1. The datasets generated by the survey may be made available upon request by following the appropriate procedure. Any member of PROGRAMMING can propose research or policy questions to be explored within the survey data following a specific set of recommendations on how to access them. We have created 2 proposal forms for the analyses of data, which are published on the PROGRAMMING website and available in Multimedia Appendices 10 and 11.

A review committee will assess each proposal and decide whether access to the data should be granted based on the following criteria: expertise of the leading proponents, relevance of the proposal’s question, informative background and convincing rationale, soundness of the proposed methods, significance of the expected novelty or impact, and feasibility of the proposed timeline. We encourage every member of PROGRAMMING to submit a specific and structured proposal form to the review committee, which will supervise and coordinate the various proposals, to promote cooperation and prevent duplication of efforts. New applications to join the European Cooperation in Science and Technology Action PROGRAMMING CA21122 are possible for the entire duration of the Action, and the process for this it outlined on the Action website [25,26].
