# Supplementary material for: Educational Needs in Geriatric Medicine Among Health Care Professionals and Medical Students in COST Action 21122 PROGRAMMING: Mixed-Methods Survey Protocol
Source: JMIR Res Protoc. 2025 Jun 3;14:e64985. doi: 10.2196/64985 (PMC12174867; doi:10.2196/64985)
Supplement: Multimedia Appendix 1 [file resprot_v14i1e64985_app1.pdf]

Multimedia Appendix 1: the PROGRAMMING survey on educational needs

Please, find hereafter the original and complete survey questionnaire in English language of the survey on educational interests and needs of healthcare professionals and final-year medical students by the COST Action PROmoting GeRiAtric Medicine in countries where it is still eMergING (PROGRAMMING) CA21122.

Thank you very much for reading it.

PROGRAMMING COST Action 21122

INFORMED CONSENT

This survey aims to map the educational interests and needs of current and future healthcare professionals in the field of care for older people. This survey is part of the European Cooperation in Science and Technology (COST) Action "**PROmoting GeRiAtric Medicine in countries where it is still eMergING**" (**PROGRAMMING**), CA21122. COST Actions are funded by the European Union. For more information, please visit: www.cost.eu and https://cost-programming.eu/

COST (European Cooperation in Science and Technology) is a funding agency for research and innovation networks. COST Actions help connect research initiatives across Europe and enable scientists to grow their ideas by sharing them with their peers. This boosts their research, career and innovation.

The European Geriatric Medicine Society (EuGMS) is Grant Holder of PROGRAMMING, CA21122.

This survey can be filled in by:

- Medical students (in the final year of the School of Medicine)
- Medical Doctors in training
- Medical Doctors (not in training)
- Nurses
- Physiotherapists
- Occupational therapists
- Speech and language therapists
- Art therapists
- Podiatrists / chiropodists
- Radiographers
- Dieticians
- Dentists
- Dental technicians

- Psychologists or psychotherapists
- Pharmacists
- Nurse assistants or healthcare assistants
- Policymakers or Public Health professionals
- Educationalists
- Professionals at the Ministry of Health, Ministry of Education or other Ministries relevant to the care of older people
- Researchers
- Social workers
- Other healthcare professionals
- Managers in the healthcare sector

Disclaimer If you continue, you are giving your informed consent to this survey and you are accepting the EuGMS privacy policy.
The EuGMS is compliant with the General Data Protection Regulation (GDPR) (EU) 2016/679. To learn more about how the EuGMS collects, keeps, and processes private information in compliance with GDPR, please visit: https://www.eugms.org/legal-and-ethical-issues/privacy-policy.html

In case you wish to receive information and/or educational material relevant to the PROGRAMMING COST Action and the EuGMS's activities you may provide us with your email (optional). [This sentence was deleted in the German version of the survey that was disseminated in Germany, in line with local Ethics regulations]

For further information about this survey, please contact: secretary@eugms.org
This survey takes about 10-15 minutes.

Section 1: Demographics

1.1 Gender*

You are:

- [1] Man
- [2] Woman
- [3] Prefer not to say

1.2 Age*

You are: *[Drop-down list]*

1. 16 years old
2. 17 years old
3. …

84. 99 years old
85. aged 100 years or older
86. Prefer not to say

1.3 Role or profession*

Which one option best describes your role or profession at present? If you have two or more roles or professions, please, select "other" and specify them.

At present you are a / work as (only one option):

- [1] Art therapist
- [2] Dental technician
- [3] Dentist
- [4] Dietician
- [5] Educationalist
- [6] Manager in the healthcare sector
- [7] Medical student (in the final year of the School of Medicine)

- [8] Medical Doctor in training
- [9] Medical Doctor (not in training)
- [10] Nurse
- [11] Nurse assistant or healthcare assistant
- [12] Occupational therapist
- [13] Pharmacist
- [14] Physiotherapist
- [15] Podiatrist / chiropodist
- [16] Policymaker or Public Health professional
- [17] Professional at the Ministry of Health or Education or other Ministry relevant to the care of older people
- [18] Psychologist or psychotherapist
- [19] Radiographer
- [20] Researcher
- [21] Social worker
- [22] Speech and Language therapist
- [23] Other healthcare professional
- [24] Other:………………

Section 2: Topics and skills

In this section, we would like to know more about:

1) your knowledge / competence of major geriatric topics and skills (i.e. how familiar you are with the topic / how confident you feel in performing the skills such as assessment);
2) their relevance to your current or future work or clinical practice; and
3) your interest in receiving further education / training on them.

Please, note that we are referring to your CURRENT knowledge and competence, which you acquired through lifetime learning, including pre-graduate and post-graduate education, formal and informal learning.

2.1 Healthy ageing and health promotion*

	[1] Very low	[2] Low	[3] Fair	[4] High	[5] Very high
Knowledge / competence					
Relevance to your work / practice					
Your interest in receiving additional education / training on this topic					

2.2 Resilience and diversity of the older person*

Awareness of these concepts: 1) the heterogeneity of ageing (both between people and among organ systems within one person); 2) the resilience with ageing, and how adaptation to change is correlated with successful ageing; 3) cohort (or generation) effects related to the events/values/experiences of the time period during which the older person matured; and 4) how multiple medical comorbidities or sensory impairments may impact the evaluation of the older person.

	[1] Very low	[2] Low	[3] Fair	[4] High	[5] Very high
Knowledge / competence					

Relevance to your work / practice					
Your interest in receiving additional education / training on this topic					

2.3 Biology of ageing*

Understanding the cellular and molecular processes underlying age-related changes as well as those accompanying the onset of age-related diseases.

	[1] Very low	[2] Low	[3] Fair	[4] High	[5] Very high
Knowledge / competence					
Relevance to your work / practice					
Your interest in receiving additional education / training on this topic					

2.4 Frailty (or related concept such as Gerastenia or intrinsic capacity)*

Frailty is a clinical syndrome of increased vulnerability, resulting from age-associated decline in reserve and function across multiple physiologic systems, so that minor stressors may lead to adverse outcomes.

	[1] Very low	[2] Low	[3] Fair	[4] High	[5] Very high
Knowledge / competence					
Relevance to your work / practice					
Your interest in receiving additional education / training on this topic					

2.5 Comprehensive Geriatric Assessment (CGA)*

CGA is the multi-disciplinary and multi-dimensional assessment and identification of medical, functional, mental, social, and environmental problems that affect the older person’s health and the

formulation and delivery of a care plan of interventions, including rehabilitation, around patient-centred goals.

	[1] Very low	[2] Low	[3] Fair	[4] High	[5] Very high
Knowledge / competence					
Relevance to your work / practice					
Your interest in receiving additional education / training on this topic					

2.6 Falls and mobility*

Recognizing and managing an older person's risk of falls, based on multifactorial falls risk assessment (including history and assessment of gait and balance, polypharmacy, co-morbidities, orthostatic hypotension and others)

	[1] Very low	[2] Low	[3] Fair	[4] High	[5] Very high
Knowledge / competence					
Relevance to your work / practice					
Your interest in receiving additional education / training on this topic					

2.7 Bone health: osteoporosis*

	[1] Very low	[2] Low	[3] Fair	[4] High	[5] Very high
Knowledge / competence					
Relevance to your work / practice					
Your interest in receiving additional education / training on this topic					

2.8 Sarcopenia*

Sarcopenia is the loss of skeletal muscle mass, strength and function.

	[1] Very low	[2] Low	[3] Fair	[4] High	[5] Very high
Knowledge / competence					
Relevance to your work / practice					
Your interest in receiving additional education / training on this topic					

2.9 Delirium (state of acute confusion)*

	[1] Very low	[2] Low	[3] Fair	[4] High	[5] Very high
Knowledge / competence					
Relevance to your work / practice					
Your interest in receiving additional education / training on this topic					

2.10 Depression or other affective disorder in older people*

	[1] Very low	[2] Low	[3] Fair	[4] High	[5] Very high
Knowledge / competence					
Relevance to your work / practice					
Your interest in receiving additional education / training on this topic					

2.11 Mild cognitive impairment and dementia: screening and differential diagnosis*

	[1] Very low	[2] Low	[3] Fair	[4] High	[5] Very high
Knowledge / competence					

Relevance to your work / practice					
Your interest in receiving additional education / training on this topic					

2.12 Dementia: management of behavioural and psychological symptoms*

	[1] Very low	[2] Low	[3] Fair	[4] High	[5] Very high
Knowledge / competence					
Relevance to your work / practice					
Your interest in receiving additional education / training on this topic					

2.13 Assessing the cognitive status of the older person using the Mini Mental State Examination (MMSE) or other cognitive screening tool*

	[1] Very low	[2] Low	[3] Fair	[4] High	[5] Very high
Knowledge / competence					
Relevance to your work / practice					
Your interest in receiving additional education / training on this topic					

2.14 Assessing the functional capacity of the older person, for example using Basic Activities of Daily Living (ADLs) and Instrumental ADLs (IADLs) scales or Barthel Scale*

	[1] Very low	[2] Low	[3] Fair	[4] High	[5] Very high
Knowledge / competence					
Relevance to your work / practice					

cost EUROPEAN COOPERATION IN SCIENCE & TECHNOLOGY
Funded by the European Union
EuGMS European Geriatric Medicine Society
Fostering geriatric medicine across Europe

Your interest in receiving additional education / training on this topic					

2.15 Assessing the capacity of the older person to consent to decisions related to their health and healthcare treatments*

	[1] Very low	[2] Low	[3] Fair	[4] High	[5] Very high
Knowledge / competence					
Relevance to your work / practice					
Your interest in receiving additional education / training on this topic					

2.16 Advance healthcare directives and planning*

They are directives in which a person specifies what actions should or should not be taken for their health (living will) or who is authorised to make decisions on their behalf (power of attorney or healthcare proxy) when they are no longer able to make decisions for themselves due to illness or incapacity.

	[1] Very low	[2] Low	[3] Fair	[4] High	[5] Very high
Knowledge / competence					
Relevance to your work / practice					
Your interest in receiving additional education / training on this topic					

2.17 Palliative care / End of Life care*

	[1] Very low	[2] Low	[3] Fair	[4] High	[5] Very high
Knowledge / competence					
Relevance to your work / practice					

Your interest in receiving additional education / training on this topic					

2.18 Persistent or chronic pain in the older person*

	[1] Very low	[2] Low	[3] Fair	[4] High	[5] Very high
Knowledge / competence					
Relevance to your work / practice					
Your interest in receiving additional education / training on this topic					

2.19 Geriatric rehabilitation*

	[1] Very low	[2] Low	[3] Fair	[4] High	[5] Very high
Knowledge / competence					
Relevance to your work / practice					
Your interest in receiving additional education / training on this topic					

2.20 Orthogeriatrics*

The multidisciplinary management and rehabilitation of the older person with low-trauma "fragility" fractures such as hip fractures and non-hip fractures, following surgery or other treatments

	[1] Very low	[2] Low	[3] Fair	[4] High	[5] Very high
Knowledge / competence					
Relevance to your work / practice					
Your interest in receiving additional education / training on this topic					

2.21 Oncogeriatrics (the care of the older person with cancer)*

	[1] Very low	[2] Low	[3] Fair	[4] High	[5] Very high
Knowledge / competence					
Relevance to your work / practice					
Your interest in receiving additional education / training on this topic					

2.22 Gerodontology*

Including: 1) performing an initial oral health assessment in older people, 2) assessing the impact of medications on the oral health of older people, 3) deciding when to refer to a dentist, and 4) demonstrating oral hygiene measures to older people and their carers.

	[1] Very low	[2] Low	[3] Fair	[4] High	[5] Very high
Knowledge / competence					
Relevance to your work / practice					
Your interest in receiving additional education / training on this topic					

2.23 Nutritional assessment and management of malnutrition of the older person*

Including: 1) identifying older people who are malnourished or at risk of malnutrition using the Mini Nutritional Assessment – Short Form or other screening tool and 2) knowing when to refer to a dietician or when to prescribe nutritional supplements.

	[1] Very low	[2] Low	[3] Fair	[4] High	[5] Very high
Knowledge / competence					
Relevance to your work / practice					

Your interest in receiving additional education / training on this topic					

2.24 Assessment and management of swallowing issues (dysphagia)*

Including: 1) identifying older people with swallowing difficulties and 2) knowing when to refer to a speech and language therapist and 3) knowing risks and benefits of feeding options.

	[1] Very low	[2] Low	[3] Fair	[4] High	[5] Very high
Knowledge / competence					
Relevance to your work / practice					
Your interest in receiving additional education / training on this topic					

2.25 Polypharmacy and deprescribing*

Including: 1) medication review and optimising drug treatment; 2) promoting the older person's adherence to drug treatment; 3) deprescribing using deprescribing tools such as STOPP START. Deprescribing is the planned and supervised process of intentionally stopping a medication or reducing its dose to optimise medication use and manage unnecessary polypharmacy.

	[1] Very low	[2] Low	[3] Fair	[4] High	[5] Very high
Knowledge / competence					
Relevance to your work / practice					
Your interest in receiving additional education / training on this topic					

2.26 Urinary and faecal incontinence*

Including diagnosis, assessment, management and preventive strategies for urinary and faecal incontinence.

	[1] Very low	[2] Low	[3] Fair	[4] High	[5] Very high
Knowledge / competence					
Relevance to your work / practice					
Your interest in receiving additional education / training on this topic					

2.27 Hearing and vision impairments of the older person*

	[1] Very low	[2] Low	[3] Fair	[4] High	[5] Very high
Knowledge / competence					
Relevance to your work / practice					
Your interest in receiving additional education / training on this topic					

2.28 Skin care and pressure ulcers*

Diagnosing, managing and preventing skin damage and pressure ulcers.

	[1] Very low	[2] Low	[3] Fair	[4] High	[5] Very high
Knowledge / competence					
Relevance to your work / practice					
Your interest in receiving additional education / training on this topic					

2.29 Ageism*

Being aware of, recognising and managing prejudice or discrimination on the grounds of a person's age.

	[1] Very low	[2] Low	[3] Fair	[4] High	[5] Very high
Knowledge / competence					

Relevance to your work / practice					
Your interest in receiving additional education / training on this topic					

2.30 Abuse of older people*

Recognising abuse of older people and knowing how to refer to appropriate services.

	[1] Very low	[2] Low	[3] Fair	[4] High	[5] Very high
Knowledge / competence					
Relevance to your work / practice					
Your interest in receiving additional education / training on this topic					

2.31 Assessing and managing the older person presenting to the Emergency Department*

	[1] Very low	[2] Low	[3] Fair	[4] High	[5] Very high
Knowledge / competence					
Relevance to your work / practice					
Your interest in receiving additional education / training on this topic					

2.32 Communication with the older person and their families, caregivers or proxies*

	[1] Very low	[2] Low	[3] Fair	[4] High	[5] Very high
Knowledge / competence					
Relevance to your work / practice					

Your interest in receiving additional education / training on this topic					

2.33 Communication and working within a multidisciplinary team*

A multidisciplinary team is a group of healthcare professionals, from different disciplines, such as nurses, doctors, administrators and others, who work together as a team to deliver comprehensive patient care

	[1] Very low	[2] Low	[3] Fair	[4] High	[5] Very high
Knowledge / competence					
Relevance to your work / practice					
Your interest in receiving additional education / training on this topic					

2.34 Overall knowledge and competence in care for older people*

How would you rate your overall knowledge and competence in care of older people, in relation to what is needed for your role or profession?

- [1] Very poor
- [2] Poor
- [3] Fair
- [4] Good
- [5] Very good

Section 3 Medical students versus professionals

Medical students versus professionals*

You are (if you are a retired or unemployed professional or a professional other than a healthcare professional, please, select "a professional"):

- [1] A medical student (in the final year of the School of Medicine) [go to Section 6, then 7 and 8]
- [2] A professional (e.g. Medical Doctor in training, Medical Doctor not in training, nurse, physiotherapist, researcher,...) [go to Section 4 and then 5 and then 7 and 8]

Section 4: Current profession (PROFESSIONALS)

PLEASE, COMPLETE ONLY IF YOU ARE A PROFESSIONAL. IF YOU ARE A MEDICAL STUDENT, PLEASE GO BACK TO SECTION 3

4.1 Main qualification or degree*

Please, select the **main** qualification or degree you hold (only one option).

- [1] Degree in Art Therapy
- [2] Degree in Dental techniques
- [3] Degree in Dentistry
- [4] Degree in Diagnostic Radiography
- [5] Degree in Economy
- [6] Degree in Law
- [7] Degree in Medicine (without further postgraduate training)
- [8] Degree in Medicine (with further postgraduate training)
- [9] Degree in Nursing Sciences
- [10] Degree in Nutritional Sciences
- [11] Degree in Occupational Therapy
- [12] Degree in Pharmacy
- [13] Degree in Philosophy
- [14] Degree in Physiotherapy
- [15] Degree in Podiatry
- [16] Degree in Political Sciences
- [17] Degree in Psychology
- [18] Degree in Psychotherapy
- [19] Degree in Public Health
- [20] Degree in Speech and Language Therapy
- [21] Qualification as nurse assistant or nurse practitioner or nurse associate or healthcare assistant

- [22] Other healthcare degree
- [23] Other non-healthcare degree
- [24] Other:………………………….

4.2 Year when main qualification or degree was obtained*

Please, tell us when you obtained your **main** qualification or degree (for Medical Doctors, with or without further training, this the year when they obtained their **Degree in Medicine**). *[Drop-down list]*

1. 1945
2. 1946
3. …

…..

79. 2023
80. 2024
81. 2025
82. 2026

4.3 Country or main country where you have STUDIED for your main qualification or degree*

Please, tell us where you obtained your **main** qualification or degree (for Medical Doctors, with or without further training, this the country where they obtained their **Degree in Medicine**). *[Drop-down list, in alphabetical order]*

1. Afghanistan
2. Albania
3. Algeria
4. ….

194. Zimbabwe
195. Other country or more than one country

4.4 Medical Specialty*

You are / You have a Medical Specialty in:

If you are not a Medical Doctor or you are a Medical Doctor in training or without further postgraduate training, please select the correct “not applicable” option. If you have a Medical Specialty that is not listed, please, select "other Medical Specialty (not in the list)". If you have more than one Medical Specialty, please, select “other (more than one Medical Specialty)”.

[Drop-down list]

1. Not applicable: Not a Medical Doctor, I am another professional
2. Not applicable: Medical Doctor in training / Medical Doctor without further postgraduate training
3. Accident and emergency medicine
4. Allergology
5. Anaesthetics
6. Cardiology
7. Child psychiatry
8. Clinical biology
9. Clinical chemistry
10. Clinical microbiology
11. Clinical neurophysiology
12. Craniofacial surgery
13. Dermatology
14. Endocrinology
15. Family and General Medicine
16. Gastroenterologic surgery
17. Gastroenterology
18. General Practice
19. General surgery
20. Geriatric Medicine
21. Haematology

22. Immunology
23. Infectious diseases
24. Internal medicine
25. Laboratory medicine
26. Nephrology
27. Neuropsychiatry
28. Neurology
29. Neurosurgery
30. Nuclear medicine
31. Obstetrics and gynaecology
32. Occupational medicine
33. Oncology
34. Ophthalmology
35. Oral and maxillofacial surgery
36. Orthopaedics
37. Otorhinolaryngology
38. Paediatric surgery
39. Paediatrics
40. Pathology
41. Pharmacology
42. Physical medicine and rehabilitation
43. Plastic surgery
44. Podiatric surgery
45. Preventive medicine
46. Psychiatry
47. Public health
48. Radiation Oncology
49. Radiology
50. Respiratory medicine

51. Rheumatology
52. Stomatology
53. Thoracic surgery
54. Tropical medicine
55. Urology
56. Vascular surgery
57. Venereology
58. Other Medical Specialty (not in the list)
59. Other (more than one Medical Specialty)

4.5 Year when Medical Specialty was obtained*

If you are not a Medical Doctor or you are a Medical Doctor in training or without further postgraduate training, please select "not applicable". If you have more than one Medical Specialty, please, select "other".

[Drop-down list]

1. Not applicable (I am not a Medical Doctor, or I am a Medical Doctor in training or a Medical Doctor without further postgraduate training)
2. 1950
3. 1951
4. ….

78. 2026
79. Other (more than one Medical Specialty)

4.6 Country or main country where you have STUDIED for your Medical Specialty*

If you are not a Medical Doctor or you are a Medical Doctor still in training or without further postgraduate training, please select "**not applicable**". If you have obtained one or more Medical Specialty in more than one country, please, select "**other**".

[Drop-down list]

1. Not applicable (I am not a Medical Doctor, or I am a Medical Doctor in training or without further postgraduate training)
2. Afghanistan
3. Albania
4. Algeria
5. …..

195. Zimbabwe
196. Other (more than one Medical Specialty)

4.7 If you have more than one Medical Specialty or one or more Sub-Specialty*

Please, list the Medical Specialties or Sub-Specialties that you obtained, and, for each of them, the year when you obtained them and the country where you obtained them. If this question is not applicable to you, please, write "**not applicable**".

[Long-answer text; maximum character count: 700]

4.8 Are you a healthcare professional (other than a Medical Doctor) with a specialty or sub-specialty in care of older people?*

- Yes (e.g. dementia nurse, old age psychologist or other)
- No
- Not applicable (I am a Medical Doctor or medical student).
- Not applicable (e.g. no clinical practice)
- Other…………….

4.9 Country where you WORK at present*

Please, select the country where you work at present.

[Drop-down list]

1. Afghanistan

2. Albania
3. …
194. Zimbabwe
195. Other country or more than one country

4.10 Settings*

Please, select the setting or settings where you work at present (multiple options are possible):

- [1] GP practice (Primary care)
- [2] Dental school clinic (Primary care)
- [3] Other Primary care or Community settings (e.g. for community nurses)
- [4] Outpatient specialist clinic (Secondary care)
- [5] Acute general hospital (Secondary care)
- [6] Acute psychiatric hospital (Secondary care)
- [7] General and or specialist rehabilitation hospital (Rehabilitation setting)
- [8] Long term psychiatric hospital (Long term care)
- [9] Long term care facilities (i.e. care homes, nursing homes) (Long term care)
- [10] City council / Region / State / Ministries
- [11] Other:…………………………………………………………..

4.11 Settings: Primary care and Community*

Please, select the Primary care and Community setting where you work at present (only one option). If you do not work in any, please, select "**I do NOT work in any Primary care or Community settings**". If you work in **more than one** Primary care and Community setting, please, select "**other**" and specify.

- [1] I do NOT work in any Primary care or Community settings
- [2] GP practice
- [3] Walk-in centres
- [4] two hour rapid crisis response services

- [5] Bed-based community rehabilitation
- [6] Community end of life and palliative care
- [7] Community Geriatric Medicine (to be selected by community geriatricians)
- [8] Community occupational therapy
- [9] Community physiotherapy
- [10] Community podiatry
- [11] Community Specialist nursing (for example, to be selected by dementia nurses, mental health nurses, diabetes nurses, heart failure nurses, incontinence nurses, tissue viability nurses)
- [12] Community speech and language therapy
- [13] District nursing
- [14] Intermediate care services
- [15] Dental practice (including private, community or Dental School Clinics)
- [16] Local Pharmacy
- [17] Other……………………..

4.12 Setting: Acute hospitals (Secondary care)*

Please, select the setting within Acute hospitals (Secondary care) where you work at present (only one option). If you do not work in any, please, select "**I do NOT work in any acute hospitals (Secondary care) settings**". If you work in **more than one or other** Acute hospitals (Secondary care) setting, please, select "**other**" and specify.

- [1] I do NOT work in any Acute general hospital settings
- [2] Accident and emergency (A&E) or Casualty Department
- [3] Allergology
- [4] Anaesthetics
- [5] Anatomical pathology
- [6] Breast screening

- [7] Cardiology
- [8] Clinical Psychology
- [9] Critical care
- [10] Day care (medical)
- [11] Day care (surgical)
- [12] Dental Department
- [13] Diagnostic imaging (or Radiology or X-ray Department)
- [14] Discharge lounge
- [15] Ear nose and throat (ENT)
- [16] Endocrinology
- [17] Forensic and Legal Medicine
- [18] Gastroenterology
- [19] General surgery
- [20] Geriatric Medicine
- [21] Gynaecology
- [22] Haematology
- [23] Hospice or Palliative Medicine
- [24] Human Resources
- [25] Infective Diseases
- [26] Internal Medicine or General Medicine
- [27] IT Services
- [28] Maternity departments
- [29] Medical Direction
- [30] Microbiology
- [31] Mortuary
- [32] Neonatal unit
- [33] Nephrology
- [34] Neurology

- [35] Nutrition and dietetics
- [36] Obstetrics and gynaecology units
- [37] Occupational Health
- [38] Occupational therapy
- [39] Oncology
- [40] Ophthalmology
- [41] Orthopaedics
- [42] Paediatrics
- [43] Pain management clinics
- [44] Pharmacy
- [45] Physiotherapy
- [46] Psychiatry
- [47] Public Health
- [48] Radiotherapy
- [49] Renal unit
- [50] Respiratory Medicine or Pneumology
- [51] Rehabilitation wards
- [52] Rheumatology
- [53] Sexual health (genitourinary medicine)
- [54] Stroke Medicine
- [55] Tropical Medicine
- [56] Urology
- [57] Other…………………………………………..

4.13 Other settings*

Please, select the setting where you work at present (only one option). If you work in **more than one** of these settings, please, select "**other**" and specify.

- [1] I do NOT work in any of the Settings below
- [2] Local City Council
- [3] Region
- [4] National Parliament
- [5] Ministry of Education
- [6] Ministry of Health
- [7] Ministry of Justice
- [8] Ministry of Social Policies / Social Development
- [9] Other Ministry
- [10] World Health Organization
- [11] Other………………..

4.14 Years of experience in caring for older people*

You have:

- No professional clinical experience in caring for older people or less than 1 year
- 1 to 4 years
- 5 to 9 years
- 10 years or over
- Other…………..

4.15 Among the patients you care for, how many are older people?*

In your clinical practice, older people account for:

- [1] Not applicable (e.g. no clinical practice)
- [2] Less than 25% of my patients
- [3] Between 25% and 50% of my patients
- [4] Between 50% and 75% of my patients
- [5] More than 75% of my patients

- [6] Other………..

Section 5 Previous education in Geriatric Medicine (PROFESSIONALS)

PLEASE, COMPLETE ONLY IF YOU ARE A PROFESSIONAL. IF YOU ARE A MEDICAL STUDENT, PLEASE GO BACK TO SECTION 3

The following questions are **about your formal professional or undergraduate university education** (e.g. **undergraduate** Medical School, School of Nursing Sciences, School of Physiotherapy, School of Pharmacy, School of Psychology, School of Law,…).

5.1 Courses / Lectures in care of older people / Geriatric Medicine*

Did you attend a specific course or specific lectures in care of older people / Geriatric Medicine?

- [1] Yes
- [2] No
- [3] Don't know / not sure

5.2 Clinical rotations (or internships or clinical training)*

Did you have a clinical rotation (or internship / clinical training) in:

Please, select "no", if there were no Geriatric Medicine acute hospital wards or outpatient clinics where you had your formal professional or undergraduate university education.

	[1] Yes	[2] No	[3] Don't know / not sure
a Geriatric Medicine acute hospital ward?			
a rehabilitation setting?			
a care home or nursing home?			
a Geriatric Medicine outpatient clinic?			

5.3 Volunteer work*

Have you ever done volunteer work with older people?

- [1] Yes
- [2] No
- [3] Don’t know / not sure

5.4 Research work*

Have you ever done research work involving older people, or on ageing, age-related diseases, or pathways and needs of older people in the social and healthcare system?

- [1] Yes
- [2] No
- [3] Don’t know / not sure

Section 6 Education in Geriatric Medicine (MEDICAL STUDENTS)

PLEASE, COMPLETE ONLY IF YOU ARE A MEDICAL STUDENT. IF YOU ARE A HEALTHCARE PROFESSIONAL, PLEASE GO BACK TO SECTION 3.

During your **Medical School**, did you attend / are you going to attend the following?

6.1 Courses / Lectures in care of older people / Geriatric Medicine*

Did you attend / are you going to attend a specific course or specific lectures in care of older people / Geriatric Medicine?

- [1] Yes, done (mandatory)
- [2] Yes, done (elective)
- [3] Yes, I will (mandatory)
- [4] Yes, I will (elective)
- [5] No
- [6] Don’t know / not sure

6.2 Clinical rotations (or internships or clinical training)*

Did you have / are you going to have a clinical rotation (or internship / clinical training) in:

Please, select "no", if there are no Geriatric Medicine acute hospital wards or outpatient clinics affiliated to the Medical School you are attending.

	[1] Yes, done (mandatory)	[2] Yes, done (elective)	[3] Yes, I will (mandatory)	[4] Yes, I will (elective)	[5] No	[6] Don’t know / not sure
a Geriatric Medicine acute hospital ward?						
a rehabilitation setting?						

a care home or nursing home?						
a Geriatric Medicine outpatient clinic?						

6.3 Volunteer work*

Have you done / are you going to do volunteer work with older people?

- [1] Yes, I have done
- [2] Yes, I am going to do
- [3] No
- [4] Don’t know / not sure

6.4 Research work*

Have you done / are you going to do research work involving older people, or on ageing, age-related diseases, or pathways and needs of older people in the social and healthcare system?

- [1] Yes
- [2] No
- [3] Don’t know / not sure

6.5 Country or main country where you are STUDYING for your Degree in Medicine*

Please, tell us where you are studying for your Degree in Medicine: *[Drop-down list]*

1. Afghanistan
2. Albania
3. …

194. Zimbabwe
195. Other country or more than one country

6.6 Name of University / Universities*

Please, write the name of the University / Universities where you are studying for your Degree in Medicine. If you have attended more than one University, please, write the names of both or all of these (maximum 200 characters). If you would prefer not to write it, please, write "BLANK".

[Long-answer text; maximum character count 200]

6.7 Would you like to become a geriatrician?*

- [1] Yes, I would like to become a geriatrician
- [2] Yes, but there are obstacles (including lack of School of Specialty in Geriatric Medicine in my country; please, see next question)
- [3] Never thought about it but maybe
- [4] No
- [5] Don't know / not sure
- Other…

6.8 Obstacles to becoming a geriatrician*

Please, tell us which obstacles you may face to becoming a geriatrician (multiple options):

- [1] I do not want to become a geriatrician
- [2] There is no School of Specialty in Geriatric Medicine in my country
- [3] There is one or more School of Specialty in Geriatric Medicine in my country but I would have to move town
- [4] There is one or more School of Specialty in Geriatric Medicine in my country but there are very few posts and I may not be selected in
- [5] Financial barriers
- [6] Child care
- [7] Being a geriatrician lacks social status, compared to being another medical or surgical specialist

- [8] Being a geriatrician is not remunerative, compared to being another medical or surgical specialist
- [9] Geriatricians have a small selection of types of job opportunities in my country
- [10] Geriatricians have difficulty in finding a job in my country because there are too many geriatricians
- [11] Geriatricians have difficulty in finding a job in my country because Geriatric Medicine is a new or relatively unknown Specialty
- [12] Geriatricians have difficulty in finding a job in my country for various reasons
- [13] None of the above barriers
- [14] Don't know / not sure
- [15] Other……………………………………………..

6.9 What are your thoughts on becoming a geriatrician?*

Please, tell me more about why you would like to become a geriatrician or not, and what would be the obstacles or facilitators to become a geriatrician (maximum 700 characters). If you would prefer not to write it, please, write "BLANK".

[Long-answer text; maximum character count 700]

6.10 Being a geriatrician in your country*

How prestigious it is to be a geriatrician in your country?

- [1] There are no geriatricians in my country
- [2] Very little
- [3] Little
- [4] Fairly
- [5] Much
- [6] Very much
- [7] Don't know / not sure

Section 7: Interest in care of older people / Geriatric Medicine

7.1 Do you enjoy engaging with older people?*

Please, rate your **satisfaction and feeling comfortable** when interacting with older people in both professional and non-professional settings.

- [1] Very low
- [2] Low
- [3] Fair
- [4] High
- [5] Very high

7.2 Do you enjoy being a healthcare professional caring for older people?*

If you are a healthcare professional caring for older people, please, rate your satisfaction and feeling of being comfortable and competent in a professional setting. If you are not a healthcare professional caring for older people, please, select "not applicable".

- [1] Not applicable, I am not a healthcare professional caring for older people
- [2] Very low
- [3] Low
- [4] Fair
- [5] High
- [6] Very high
- [7] Don’t know / not sure

7.3 Thoughts on caring for older people*

Please, describe your thoughts and feelings on caring for older people (maximum 700 characters) (maximum 700 characters). If you would prefer not to write it, please, write "BLANK".

[Long-answer text; maximum character count 700]

7.4 Caring for older people in your country*

How prestigious it is to care for older people in your country?

- [1] Very little
- [2] Little
- [3] Fairly
- [4] Much
- [5] Very much
- [6] Don’t know / not sure

Section 8 Suggestions on courses in care for older people or Geriatric Medicine

If we organize a Course in care for older people or Geriatric Medicine:

8.1 Interest in courses in care for older people or Geriatric Medicine*

Would you be interested in attending courses in care for older people or Geriatric Medicine?

- [1] Very little
- [2] Little
- [3] Fairly
- [4] Much
- [5] Very much

8.2 Which type of course would you choose?*

- [1] In person
- [2] Online
- [3] Hybrid (both online and in person options)

8.3 What would be the most important barrier(s) for you to attend such a course?*

	[1] Yes	[2] No	[3] Don't know / not sure
Difficulties to leave my practice due to financial loss			
Difficulties to leave my practice due to paid leave issues			
Doubts about the feasibility of the teached skills in the settings where I work			
Doubts about the relevance of teached skills to my clinical practice			

Doubts about the necessity to improve my current knowledge and skills			
Language barrier (in case the course is in English)			
Low motivation			
Time constraints			
Other			

8.4 Other barrier(s) to attend such a course*

Please, specify other barriers to attend such a course (maximum 500 characters). If you would prefer not to write it, please, write "BLANK".

[Long-answer text; maximum character count 500]

8.5 Teaching methods*

Please, rate the effectiveness of each teaching method:

	[1] Very low	[2] Low	[3] Fair	[4] High	[5] Very high
A to Z textbook like lectures on a topic					
Teaching of clinical tools for screening/diagnosis and evaluation					
Questions' driven teaching					
Clinical cases					
Role plays					
Short presentations by the participants as a learning tool					

Assignment of group activities					

8.6 Other teaching methods*

Would you suggest any other teaching methods, please? Please, specify (maximum 500 characters). If you would prefer not to write it, please, write "BLANK".

[Long-answer text; maximum character count 500]

THANK YOU FOR COMPLETING THE SURVEY

In case you wish to provide us with your email (optional), you will receive information and/or educational material relevant to the PROGRAMMING COST Action and the EuGMS's activities.

If you wish, please, write your mail (optional)

[short-answer text]

[The option to write the contact mail was deleted in the German version of the survey that was disseminated in Germany, in compliance with local Ethics regulations]

[Please, note the four questions (4.3; 4.6; 4.9; 6.5) in the survey have a drop-down list of countries as response options. However, the numbering of countries varies between these, as the first response option of question 4.6 is "not applicable". In other words, there are 195 response options for 4.3; 4.9 and 6.5; there are 196 response options for 4.6]

[The asterisk next to each question indicates that a response is required in order to continue with the survey]

[Everything that is written in italics, in square brackets is not part of the survey; it is only meant to guide a possible translation or adaptation of the survey]
