# Supplementary material for: Educational Needs in Geriatric Medicine Among Health Care Professionals and Medical Students in COST Action 21122 PROGRAMMING: Mixed-Methods Survey Protocol
Source: JMIR Res Protoc. 2025 Jun 3;14:e64985. doi: 10.2196/64985 (PMC12174867; doi:10.2196/64985)
Supplement: Multimedia Appendix 2 [file resprot_v14i1e64985_app2.docx]

**Multimedia Appendix 2: Composition of the international panel of stakeholders (online meeting, February 22, 2023)**

| **ID** | **Country / Region** | **Professional group** |
| --- | --- | --- |
| **TM** | United Kingdom | Physicians and Surgeons |
| **GO** | United Kingdom | Physicians and Surgeons |
| **1** | Belgium | Physicians and Surgeons |
| **2** | Belgium | Physiotherapists |
| **3** | Bulgaria | Physicians and Surgeons |
| **4** | Bulgaria | Physiotherapists |
| **5** | Croatia | Medical students |
| **6** | Croatia | Physicians and Surgeons |
| **7** | Cyprus | Researchers |
| **8** | Czech Republic | Researchers |
| **9** | Czech Republic | Physicians and Surgeons |
| **10** | Denmark | Researchers |
| **11** | France | Physicians and Surgeons |
| **12** | France | Physicians and Surgeons |
| **13** | France | Physicians and Surgeons |
| **14** | Greece | Other healthcare professionals |
| **15** | Greece | Physicians and Surgeons |
| **16** | Greece | Educationalists or professionals at the Ministry of Education |
| **17** | Greece | Physicians and Surgeons |
| **18** | Israel | Policymakers or Public Health professionals |
| **19** | Italy | Physiotherapists |
| **20** | Italy | Managers in the healthcare sector |
| **21** | Italy | Physicians and Surgeons |
| **22** | Italy | Psychologists or psychotherapists |
| **23** | Italy | Physiotherapists |
| **24** | Italy | Nurses |
| **25** | Italy | Other healthcare professionals |
| **26** | Kosovo | Researchers |
| **27** | Kosovo | Nurses |
| **28** | Kosovo | Policymakers or Public Health professionals |
| **29** | Latvia | Researchers |
| **30** | Latvia | Physiotherapists |
| **31** | Latvia | Occupational therapists |
| **32** | Lithuania | Physicians and Surgeons in training |
| **33** | Malta | Physicians and Surgeons |
| **34** | Malta | Managers in the healthcare sector |
| **35** | Malta | Physicians and Surgeons |
| **36** | Malta | Physicians and Surgeons |
| **37** | Montenegro | Nurses |
| **38** | Montenegro | Physicians and Surgeons |
| **39** | Montenegro | Physicians and Surgeons |
| **40** | Montenegro | Physicians and Surgeons |
| **41** | Netherlands | Psychologists or psychotherapists |
| **42** | North Macedonia | Physicians and Surgeons |
| **43** | North Macedonia | Physicians and Surgeons |
| **44** | North Macedonia | Physicians and Surgeons |
| **45** | North Macedonia | Physicians and Surgeons |
| **46** | North Macedonia | Nurses |
| **47** | Prefer not to say | Physiotherapists |
| **48** | Prefer not to say | Physicians and Surgeons |
| **49** | Poland | Physicians and Surgeons |
| **50** | Portugal | Physicians and Surgeons |
| **51** | Portugal | Pharmacists |
| **52** | Portugal | Dieticians |
| **53** | Portugal | Other healthcare professionals |
| **54** | Portugal | Nurses |
| **55** | Romania | Physicians and Surgeons |
| **56** | Romania | Physicians and Surgeons |
| **57** | Serbia | Educationalists or professionals at the Ministry of Education |
| **58** | Serbia | Physicians and Surgeons |
| **59** | Serbia | Physicians and Surgeons |
| **60** | Serbia | Physicians and Surgeons |
| **61** | Spain | Physiotherapists |
| **62** | Türkiye | Physicians and Surgeons |
| **63** | Türkiye | Nurses |
| **64** | Türkiye | Physicians and Surgeons |
| **65** | Türkiye | Pharmacists |
| **66** | United States | Physicians and Surgeons |
| **67** | United States | Researchers |
| **68** | United States | Physiotherapists |

Abbreviations: ID: identifier; TM: Professor Tahir Masud; GO: Dr Giulia Ogliari. The identity of those who joined the international panel of stakeholders is kept confidential in respect of their privacy.
