# Supplementary material for: Educational Needs in Geriatric Medicine Among Health Care Professionals and Medical Students in COST Action 21122 PROGRAMMING: Mixed-Methods Survey Protocol
Source: JMIR Res Protoc. 2025 Jun 3;14:e64985. doi: 10.2196/64985 (PMC12174867; doi:10.2196/64985)
Supplement: Multimedia Appendix 3 [file resprot_v14i1e64985_app3.docx]

**Multimedia Appendix 3: Sections of the PROmoting GeRiAtric Medicine in countries where it is still eMergING (PROGRAMMING) survey**

| Title of section | Questions | Respondents |
| --- | --- | --- |
| Informed consent | - Informed consent | All |
| Section 1—demographics | - Gender (question 1.1) - Age (question 1.2) - Role or profession (question 1.3) | All |
| Section 2—topics and skills | - Healthy ageing and health promotion (question 2.1) - Resilience and diversity of the older person (question 2.2) - Biology of ageing (question 2.3) - Frailty (or related concepts, such as gerastenia or intrinsic capacity; question 2.4) - CGA^a^ (question 2.5) - Falls and mobility (question 2.6) - Bone health—osteoporosis (question 2.7) - Sarcopenia (question 2.8) - Delirium (state of acute confusion; question 2.9) - Depression or other affective disorders in older people (question 2.10) - Mild cognitive impairment and dementia—screening and differential diagnosis (question 2.11) - Dementia—management of behavioral and psychological symptoms (question 2.12) - Assessing the cognitive status of the older person using the MMSE^b^ or other cognitive screening tool (question 2.13) - Assessing the functional capacity of the older person, for example, using basic ADL^c^ and IADL^d^ scales or the Barthel scale (question 2.14) - Assessing the capacity of the older patient to consent to decisions related to their health and health care treatments (question 2.15) - Advance health care directives and planning (question 2.16) - Palliative or end-of-life care (question 2.17) - Persistent or chronic pain in the older person (question 2.18) - Geriatric rehabilitation (question 2.19) - Orthogeriatrics (question 2.20) - Oncogeriatrics (the care of the older person with cancer; question 2.21) - Gerodontology (question 2.22) - Nutritional assessment and management of malnutrition of the older person (question 2.23) - Assessment and management of swallowing issues (dysphagia; question 2.24) - Polypharmacy and deprescribing (question 2.25) - Urinary and fecal incontinence (question 2.26) - Hearing and vision impairments of the older person (question 2.27) - Skin care and pressure ulcers (question 2.28) - Ageism (question 2.29) - Abuse of older people (question 2.30) - Assessing and managing the older person presenting to the emergency department (question 2.31) - Communication with the older person and their families, caregivers, or proxies (question 2.32) - Communication and working within a multidisciplinary team (question 2.33) - Overall knowledge on and competence in the care for older people (question 2.34) | All |
| Section 3—medical students versus professionals | - Question to divide medical students and professionals | All |
| Section 4—current profession (professionals) | - Main qualification or degree (question 4.1) - Year when main qualification or degree was obtained (question 4.2) - Country or main country where they studied for their main professional qualification or degree (question 4.3) - Medical specialty (question 4.4) - Year when the medical specialty was obtained (question 4.5) - Country or main country where they studied for their medical specialty (question 4.6) - Whether they had more than one medical specialty or one or more subspecialties (question 4.7) - Whether they were a health care professional (other than a medical doctor) with a specialty or subspecialty or competency in the care of older people (question 4.8) - Country where they worked at present (question 4.9) - Settings (question 4.10) - Setting—primary care and community (question 4.11) - Setting—acute care hospitals (secondary care; question 4.12) - Other settings (question 4.13) - Years of experience caring for older people (question 4.14) - Among the patients they cared for, how many were older people (question 4.15) | Professionals |
| Section 5—previous education in geriatric medicine (professionals) | - Courses or lectures on the care of older people or geriatric medicine (question 5.1) - Clinical rotations (or internships or clinical training; question 5.2) - Volunteer work (question 5.3) - Research work (question 5.4) | Professionals |
| Section 6—education in geriatric medicine (medical students) | - Courses or lectures on the care of older people or geriatric medicine (question 6.1) - Clinical rotations (or internships or clinical training; question 6.2) - Volunteer work (question 6.3) - Research work (question 6.4) - Country or main country where they were studying for their degree in medicine (question 6.5) - Name of the university or universities (question 6.6) - Whether they would like to become geriatricians (question 6.7) - Obstacles to becoming a geriatrician (question 6.8) - What were their thoughts on becoming a geriatrician (question 6.9) - Being a geriatrician in their country (question 6.10) | Final-year medical students |
| Section 7—interest in the care of older people or geriatric medicine | - Whether they enjoyed engaging with older people (question 7.1) - Whether they enjoyed being health care professionals caring for older people (question 7.2) - Thoughts on caring for older people (question 7.3) - Caring for older people in their country (question 7.4) | All |
| Section 8—suggestions on courses on the care of older people or geriatric medicine | - Interest in courses on the care of older people or geriatric medicine (question 8.1) - The type of course they would choose (question 8.2) - The most important barriers to them attending such a course (question 8.3) - Other barriers to attending such a course (question 8.4) - Teaching methods (question 8.5) | All |
| Final section | - Thank you for completing the survey | All |

^a^CGA: comprehensive geriatric assessment.

^b^MMSE: Mini-Mental State Examination.

^c^ADL: activity of daily living.

^d^IADL: instrumental ADL.
