# Supplementary material for: Educational Needs in Geriatric Medicine Among Health Care Professionals and Medical Students in COST Action 21122 PROGRAMMING: Mixed-Methods Survey Protocol
Source: JMIR Res Protoc. 2025 Jun 3;14:e64985. doi: 10.2196/64985 (PMC12174867; doi:10.2196/64985)
Supplement: Multimedia Appendix 4 [file resprot_v14i1e64985_app4.docx]

**Multimedia Appendix 4: Translation guidelines**

| **Number of rule** | **Full text** |
| --- | --- |
| **1** | **The survey must only be directly translated from English into each one of other languages.** |
| **2** | **Each translation process requires at least a translator and at least a proof-reader.** |
| **3** | The order of questions and responses must be kept. |
| **4** | No question or response can be deleted or added. |
| **5** | A few questions have lists of professions, medical specialties, settings, or countries as response options; these lists follow the English alphabetical order and translators have to keep the original English order. |
| **6** | In case a profession or medical specialty does not exist in the translated language or country of dissemination, the translator has to keep the word in English and, next to it, explain its meaning and that this is not present in their language or country. [For example, the medical specialty of Geriatric Medicine is kept in the list also in those countries that do not recognise it. Similarly, the profession of art therapist and podiatrist. In case the translator is concerned that "podiatrist" could be confused with "paediatrician", they are allowed to explain the meaning of “podiatrist” next to it. |
| **7** | The translators have to keep the words in capital letters and those in bold characters, as in the original English version. |
| **8** | The translators have to keep the drop-down list for age to avoid spelling mistakes of the respondents. |
| **9** | All of the above is done to keep the survey harmonized across countries. |
| **10** | The translators are allowed to use both the feminine and masculine words for professions, in case their language requires this. |
| **11** | The translators are allowed to translate "Comprehensive Geriatric Assessment (CGA)" into their own language but are encouraged to keep the English phrase and abbreviation in English in brackets next to it. |

A member of WG1 (GO) supervised the translations and was available to answer any queries on the translation, the structure of the survey or the management of the online survey forms. GO had online and phone discussions with the translation teams who requested these, and shared the learning points from these with all the other translations teams through emails. GO used to send the same mails of recommendations and instructions to all teams in open copy so that everyone could reply to all as in a forum.
