# Supplementary material for: Educational Needs in Geriatric Medicine Among Health Care Professionals and Medical Students in COST Action 21122 PROGRAMMING: Mixed-Methods Survey Protocol
Source: JMIR Res Protoc. 2025 Jun 3;14:e64985. doi: 10.2196/64985 (PMC12174867; doi:10.2196/64985)
Supplement: Multimedia Appendix 5 [file resprot_v14i1e64985_app5.docx]

**Multimedia Appendix 5: Email to the stakeholders from Belgium [the end date of dissemination of the survey was subsequently postponed]**

**PROGRAMMING COST Action 21122**

Dear Madam, dear Sir,

Hope this mail finds you well.

We would be very grateful if you would consider joining us in our initiative to improve the care of older people.

We have developed a survey on the educational needs of final-year medical students and healthcare professionals in the field of care for older people.

We would like to ask you to disseminate this survey among the final-year medical students of your University / members of your society, by forwarding these links, please:

to the survey in the French language: **LINK**

to the survey in the Dutch language: **LINK**

This survey is part of the European Cooperation in Science and Technology (COST) Action “PROmoting GeRiAtric Medicine in countries where it is still eMergING” (PROGRAMMING), CA21122. COST Actions are funded by the European Union. Any person with an affiliation in a legal entity can ask to join the PROGRAMMING COST Action. For more information, please refer yourself to the Action's webpage. The European Geriatric Medicine Society (EuGMS) is Grant Holder of PROGRAMMING, CA21122. It is compliant with the General Data Protection Regulation (GDPR) (EU) 2016/679.

This survey is directed to final-year medical students, healthcare professionals (including Medical Doctors in training, Medical Doctors (not in training), Nurses, Physiotherapists, Occupational therapists, Speech and language therapists, Art therapists, Podiatrists / chiropodists, Radiographers, Dieticians, Dentists, Dental technicians, Psychologists or psychotherapists, Pharmacists, Nurse assistants or healthcare assistants, Policymakers or Public Health professionals, Educationalists, Professionals at the Ministry of Health, Ministry of Education or other Ministries relevant to the care of older people), Researchers, Social workers, Other healthcare professionals, Managers in the healthcare sector, and other professionals involved in care of older people or education in care of older people.

It takes only 10-15 minutes to complete and the feedback will be of great help to improve education in the care for older people.

It will end in February 2024.

Participation to the survey is voluntary and anonymous.

For more information about this survey, please, reply to this mail.

Thank you very much for your time and consideration. Best regards,

The PROGRAMMING COST Action's Management Committee
