# Supplementary material for: Educational Needs in Geriatric Medicine Among Health Care Professionals and Medical Students in COST Action 21122 PROGRAMMING: Mixed-Methods Survey Protocol
Source: JMIR Res Protoc. 2025 Jun 3;14:e64985. doi: 10.2196/64985 (PMC12174867; doi:10.2196/64985)
Supplement: Multimedia Appendix 6 [file resprot_v14i1e64985_app6.docx]

**Multimedia Appendix 6: Simplified email to the stakeholders from Norway [the official Poster for dissemination was attached to this email and the QR code inserted at the end of the email]**

**PROGRAMMING survey on educational needs in the care of older people**

We would like to invite you to fill in this survey on the educational needs in the care of older people. It targets **final-year medical students and all healthcare professionals** (e.g. physiotherapists, psychologists, nurses, doctors, occupational therapists, SALTs, dieticians, pharmacists,…), **researchers, policy makers and other professionals involved in the (education in the) care of older people**, across **NORWAY** and European countries.

It aims to explore your interest and educational needs in Frailty, Comprehensive Geriatric Assessment, Falls and mobility, the management of chronic Pain in older people, Orthogeriatrics, Oncogeriatrics, Dementia, Delirium, Depression, Gerodontology and many other topics.

It takes only 10-15 minutes to complete. Your feedback will help us improve the care of older people. Please, fill in the survey using this link:

**LINK**

This survey is part of the European Cooperation in Science and Technology (COST) Action “PROmoting GeRiAtric Medicine in countries where it is still eMergING” (PROGRAMMING), CA21122. COST Actions are funded by the European Union. The European Geriatric Medicine Society (EuGMS) is Grant Holder of PROGRAMMING, CA21122. It is compliant with the General Data Protection Regulation (GDPR) (EU) 2016/679.

**QR code**
