# Supplementary material for: Educational Needs in Geriatric Medicine Among Health Care Professionals and Medical Students in COST Action 21122 PROGRAMMING: Mixed-Methods Survey Protocol
Source: JMIR Res Protoc. 2025 Jun 3;14:e64985. doi: 10.2196/64985 (PMC12174867; doi:10.2196/64985)
Supplement: Multimedia Appendix 7 [file resprot_v14i1e64985_app7.docx]

**Multimedia Appendix 7: Ethical considerations**

The survey protocol received ethics approval from the Jagiellonian University ethics committee, Kraków, Poland (118.6120.61.2023; request submitted on May 26, 2023; approval by the ethics committee on June 5, 2023); the Ethics Committee for Human Research, Faculty of Medicine, Ss. Cyril and Methodius University in Skopje, Republic of North Macedonia (03-338211; dated August 7, 2023); Medical University of Graz, Austria (35-373 ex 22/23; voted and approved on September 15, 2023); the Faculty of Medicine, Health, and Life Sciences Research Ethics Committee of Queen’s University Belfast, United Kingdom (23_123; September 27, 2023); Jerusalem College of Technology, Jerusalem, Israel (014_23; September 2023); the ethics committee of the Faculty of Dental Medicine, University of Belgrade, Serbia (36/42; dated October 16, 2023); the Committee of Bioethics and Deontology, School of Medicine, National and Kapodistrian University of Athens, Athens, Greece (762; October 23, 2023); Istanbul Faculty of Medicine Clinical Research Ethics Committee, Istanbul, Türkiye (application 2023/2339; decision 26; dated December 29, 2023); and the Ethics Commission of the Faculty of Medicine of the University of Cologne, Germany (application 23-1439; letter from the Ethics Commission dated March 11, 2024). The Research Ethics Committee of “Carol Davila” University of Medicine and Pharmacy, Bucharest, Romania (12996; dated May 10, 2023); the Central Denmark Region Committees on Health Research Ethics, Denmark (request 173/2023; outcome letter dated November 10, 2023); the secretariat of the Umbria Regional Ethics Committee, Italy (email dated September 13, 2023); and the Research Ethics Committee of Rīga Stradiņš University, Latvia, stated that the study may be conducted without an approval from the committees. The ethics committee of Constantine the Philosopher University in Nitra, Slovakia, approved the project implementation and questionnaires, including documentation (UKF/370/2025/191013:006). The Ethics Committee of General Hospital “Prim. Dr. Abdulah Nakas,” Sarajevo, Bosnia and Herzegovina, approved the project implementation and questionnaires, including documentation (26-213-139/25; Sarajevo, March 3, 2025). According to the Republic of Lithuania’s Law on Ethics of Biomedical Research (May 11, 2000; VIII-1679) and the assessment of the Lithuanian Bioethics Committee, the permission of the Lithuanian Bioethics Committee was not required for this survey targeting professionals; medical students were not targeted in Lithuania. According to the Swedish Ethical Review Authority, the Act on Ethical Review of Research Involving Humans (2003:460) states that “If you have designed a study in such a way that sensitive personal data will not be collected ethical review is not required.” In Albania, ethics approval was not required for this survey with deidentified data (law 80/2015, “On Higher Education and Scientific Research in Higher Education Institutions of the Republic of Albania”). In Belgium, Bulgaria, Croatia, the Czech Republic, France, the Netherlands, Portugal, and Spain, ethics approval was waived as the survey collected data that were deidentified, there were no interventions, informed consent was obtained by proceeding with the survey, and participants were informed of the use of their data in accordance with the GDPR (EU 2016/679), ensuring compliance with the applicable regulations.
