# Supplementary material for: Educational Needs in Geriatric Medicine Among Health Care Professionals and Medical Students in COST Action 21122 PROGRAMMING: Mixed-Methods Survey Protocol
Source: JMIR Res Protoc. 2025 Jun 3;14:e64985. doi: 10.2196/64985 (PMC12174867; doi:10.2196/64985)
Supplement: Multimedia Appendix 8 [file resprot_v14i1e64985_app8.docx]

**Multimedia Appendix 8: European Geriatric Medicine Society social media metrics**

| **07/11/2023**  **Facebook:**  REACH: 2630; IMPRESSIONS: 3135; INTERACTIONS: 22  **Instagram:**  REACH: 142; IMPRESSIONS: 161; INTERACTIONS: 5  **Linkedin:**  REACH: 19; IMPRESSIONS: 1293; INTERACTIONS: 35  **X:**  REACH: 1022; INTERACTIONS: 12 | **** |
| --- | --- |
| **27/11/2023**  **Facebook:**  REACH: 634; IMPRESSIONS: 810; INTERACTIONS: 9  **Instagram:**  REACH: 165; IMPRESSIONS: 190; INTERACTIONS: 5  **Linkedin:**  REACH: 139; IMPRESSIONS: 1771; INTERACTIONS: 34  **X:**  REACH: 2678; INTERACTIONS: 32 | **** |
| **24/01/2024**  **Facebook:**  REACH: 559; IMPRESSIONS: 656; INTERACTIONS: 10  **Instagram:**  REACH: 160; IMPRESSIONS: 183; INTERACTIONS: 10  **Linkedin:**  REACH: 604; IMPRESSIONS: 931; INTERACTIONS: 23  **X:**  REACH: 585; INTERACTIONS: 6 | **** |
| **19/02/2024**  **Facebook:**  REACH: 511; IMPRESSIONS: 608; INTERACTIONS: 7  **Instagram:**  REACH: 244; IMPRESSIONS: 275; INTERACTIONS: 15  **Linkedin:**  REACH: 447; IMPRESSIONS: 935; INTERACTIONS: 19  **X:**  REACH: 700; INTERACTIONS: 8 | **** |
| **26/03/2024**  **Facebook:**  REACH: 464; IMPRESSIONS: 510; INTERACTIONS: 14  **Instagram:**  REACH: 224; IMPRESSIONS: 296; INTERACTIONS: 20  **Linkedin:**  REACH: 503; IMPRESSIONS: 803; INTERACTIONS: 14  **X:**  REACH: 283; INTERACTIONS: 1 | **** |
| **15/04/2024**  **Facebook:**  REACH: 221; IMPRESSIONS: 245; INTERACTIONS: 6  **Instagram:**  REACH: 143; IMPRESSIONS: 185; INTERACTIONS: 9  **Linkedin:**  REACH: 579; IMPRESSIONS: 1044; INTERACTIONS: 18  **X:**  REACH: 421; INTERACTIONS: 2 | **** |
| **06/08/2024**  **Facebook:**  REACH: 219; IMPRESSIONS: 263; INTERACTIONS: 11  **Instagram:**  REACH: 293; INTERACTIONS: 13  **Linkedin:**  REACH: 339; IMPRESSIONS: 607; INTERACTIONS: 18  **X:**  REACH: 261; INTERACTIONS: 3 | **** |
| **24/10/2024**  **Facebook:**  REACH: 400; INTERACTIONS: 10  **Instagram:**  REACH: 224; INTERACTIONS: 12  **Linkedin:**  REACH: 324; IMPRESSIONS: 600; INTERACTIONS: 12  **X:**  REACH: 243; INTERACTIONS: 2 | **** |
