# Supplementary material for: Educational Needs in Geriatric Medicine Among Health Care Professionals and Medical Students in COST Action 21122 PROGRAMMING: Mixed-Methods Survey Protocol
Source: JMIR Res Protoc. 2025 Jun 3;14:e64985. doi: 10.2196/64985 (PMC12174867; doi:10.2196/64985)
Supplement: Multimedia Appendix 9 [file resprot_v14i1e64985_app9.docx]

**Multimedia Appendix 9: Collaborators of the PROGRAMMING survey on educational needs**

1. Prof. Klejda Harasani, ORCID 0000-0002-1877-6662, University of Medicine of Tirana, Rruga e Dibres 371, 1005, Tirana, Albania

2. Prof. Asoc. Dr. Fatjona Kamberi, ORCID 0000-0003-4793-9384, Scientific Research Centre for Public Health, University of Vlore "Ismail Qemali", Vlore, Albania

3. Dr Elona Dybeli, ORCID 0000-0002-3212-3633, University Aleksander Xhuvani, Ismail, Zyma Street, 3001 Elbasan, Albania

4. Dr Mitilda Gugu, ORCID 0000-0002-5064-3915, Faculty of Medical Technical Sciences, University of Elbasan "Aleksander Xhuvani", Elbasan, Albania

5. Dr Brunilda Elezi, ORCID 0000-0002-6099-1163, University of Elbasan "Aleksander Xhuvani", Elbasan, Albania

6. Ms. Lilit Muradyan, ORCID 0009-0003-8002-0860, Association of HealthCare and Assistance to Older People, 225A V. Achemyan, 0005 Yerevan, Armenia

7. Dr. Artur Torosyan, ORCID 0009-0003-5758-6145, Armenian Association of Geriatrics and Gerontology, 225A V. Achemyan str., 0005 Yerevan, Armenia

8. Dr Georg Ruppe, ORCID 0009-0003-2260-5158, Austrian Interdisciplinary Platform on Ageing / OEPIA, Austria

9. Ms Carolin Herzog, ORCID 0000-0001-9706-8105 Medical University of Graz, Department of Internal Medicine, Research Unit Aging and Old Age Medicine, Auenbruggerplatz 15, 8036 Graz, Austria

10. Mrs Sonja Lindner-Rabl, ORCID 0000-0002-7794-3117, Medical University of Graz, Department of Internal Medicine, Research Unit Aging and Old Age Medicine, Auenbruggerplatz 15, 8036 Graz, Austria

11. Prof. Marian Dejaeger, ORCID 0000-0002-7289-1397, Department of Public Health and Primary Care, Gerontology and Geriatrics, KU Leuven, Leuven, Belgium

12. Dr Nermina Polimac Gorana, ORCID 0009-0001-3719-2355, General Hospital Sarajevo, Sarajevo, Bosnia and Herzegovina

13. Prof. Dr Alma Pobrić, ORCID 0000-0001-7838-0267, University of Sarajevo - Faculty of Science; Zmaja od Bosne 33-35, 71000 Sarajevo, Bosnia and Herzegovina

14. Ass. Dr Amina Tucak-Smajić, ORCID 0000-0002-5149-8860, University of Sarajevo - Faculty of Pharmacy, Department of Pharmaceutical Technology; Zmaja od Bosne 8, 71000 Sarajevo, Bosnia and Herzegovina

15. Dr Ignat Petrov, ORCID 0000-0002-4372-3351, Medical University of Sofia, University Hospital of Endocrinology, Sofia, Bulgaria

16. Dr Marie-Josiane Ntsama Essomba, ORCID 0000-0002-3417-3351, University of Yaounde I - Faculty of Medicine and Biomedical Sciences, Yaounde, Cameroon

17. Marta Martin-Carbonell, ORCID 0000-0002-6337-577X, Universidad Cooperativa de Colombia, Santa Marta, Colombia

18. Dr Selma Cvijetic Avdagic, ORCID 0000-0002-8607-5999, Institute for Medical Research and Occupational Health, 10000 Zagreb, Croatia

19. Dr Maja Ortner Hadžiabdić, ORCID 0000-0003-1578-9764, University of Zagreb, Faculty of Pharmacy and Biochemistry, Zagreb, Croatia

20. Ms Sotiria Moza, ORCID 0000-0003-1209-0560, Agecare (Cyprus) Ltd, Athalassis 41, 2221 Nicosia, Cyprus

21. Ms Marina Polycarpou, ORCID 0000-0001-5530-0500, Agecare (Cyprus) Ltd, Athalassis 41, 2221 Nicosia, Cyprus

22. Dr Hana Vaňková, ORCID 0000-0002-7506-8937, Department of Internal Medicine, Third Faculty of Medicine, Charles University and University Hospital Královské Vinohrady, Ruská 87, 10000 Praha, Czech Republic

23. Dr Lucie Břízová Tománková, ORCID 0009-0001-6815-2911, No affiliation, Czech Republic

24. Dr Lene Holst Andersen, ORCID 0000-0003-0331-3136, Aarhus University - Faculty of Health, 8000 Aarhus C, Denmark

25. Dr Lone Winther Lietzen, ORCID 0000-0002-9520-406X, Department of Geriatrics and Clinical Medicine, Aarhus University Hospital, Palle Juul-Jensens Boulevard 99, 8200 Aarhus N, Denmark

26. Ass. Prof. Helgi Kolk, ORCID 0000-0001-7860-1082, Faculty of Medicine, University of Tartu L. Puusepa str. 8 51014 Tartu, Estonia

27. Prof. Jouko Laurila, ORCID 0000-0003-1836-100X, University of Oulu, 90014 Oulu, Finland

28. Dr Dimitrije Jakovljević, ORCID 0000-0002-7619-2905, Mehiläinen Oy, Geriatric services, Helsinki, Finland

29. Dr Evelyne Liuu, ORCID 0000-0003-2758-5064, Poitiers University Hospital, 86000 Poitiers, France

30. Dr Thomas Gilbert, ORCID 0000-0001-8270-7605, Lyon-sud University Hospital; RESHAPE Inserm U1290, Lyon, France

31. Prof. Maria Cristina Polidori, ORCID 0000-0002-8881-904X, Aging Clinical Research, Department II of Internal Medicine and Center for Molecular Medicine Cologne, University of Cologne, Faculty of Medicine and University Hospital Cologne, Cologne, Germany and Cologne Excellence Cluster on Cellular Stress Responses in Ageing-Associated Diseases (CECAD), University of Cologne, Cologne, Germany

32. Dr Georgios Soulis, ORCID 0000-0002-2291-5733, Hellenic Society for the Study and Research of Ageing 9, Spetson Street 15342, Agia Paraskevi, Greece

33. Dr Oraianthi Fiste, ORCID 0000-0002-7106-4162, 3rd Department of Internal Medicine, Medical School of Athens, National and Kapodistrian University of Athens, Thoracic General Hospital of Athens "I SOTIRIA", 11527 Athens, Greece

34. Dr Eleni Moumtzi-Nakka, ORCID 0000-0002-0238-3688, 414 Army Hospital of Special Diseases - 414AhoSD, 6 Taxiarchou Veliou Str, 152 36 Athens, Greece

35. Zsuzsanna Geréb Valachiné, ORCID 0000-0001-9154-9821, Eötvös Loránd University, Psychology Institute, EMIND Integrative Neuropsychology Research Group, Budapest, Hungary

36. Dr Katie Robinson, ORCID 0000-0003-1008-9857, University of Limerick, PESS, Limerick Ireland

37. Dr Ruth Usher, ORCID 0000-0001-9405-7080, University College Cork, Brookfield Health Sciences Complex, T12 AK54 Cork, Ireland

38. Prof. Anna Zisberg, ORCID 0000-0002-8234-5218, University of Haifa, 31905 Haifa, Israel

39. Rachel Kessel, No ORCID, No affiliation, Israel

40. Dr Sigal P. Naim, 0000-0003-4122-6789, Academic Center for Law and Science, Margoa st. 5, 4510201 Hod Hasharon, Israel

41. Dr Stefano Eleuteri, ORCID 0000-0002-8416-2845, Università degli Studi di Roma “La Sapienza”, Viale di Grottarossa 1035, 00199 Rome, Italy

42. Prof. Carmelinda Ruggiero, ORCID 0000-0002-1245-1963, Department of Medicine and Surgery, Gerontology and Geriatric Section, University of Perugia, 06153 Perugia

43. Premtim Rashiti, ORCID 0000-0002-9156-9213, University of Prishtina Hasan Prishtina, Str. George Bush, No.31, 10000 Prishtina, Kosovo* (*This designation is without prejudice to positions on status, and is in line with UNSCR 1244/1999 and the ICJ Opinion on the Kosovo declaration of independence)

44. Qendrese Daka, ORCID 0000-0001-8765-2973 Department of Pathophysiology, Medical Faculty, University of Prishtina, 10000 Prishtina, Kosovo* and Department of Ophthalmology, University Clinical Centre of Kosova, Prishtina, Kosovo* (*This designation is without prejudice to positions on status, and is in line with UNSCR 1244/1999 and the ICJ Opinion on the Kosovo declaration of independence)

45. Naim Jerliu, ORCID 0000-0003-4919-5454, Faculty of Medicine, University of Prishtina "Hasan Prishtina" and National Institute of Public Health of Kosovo, 10000 Prishtina, Kosovo* (*This designation is without prejudice to positions on status, and is in line with UNSCR 1244/1999 and the ICJ Opinion on the Kosovo declaration of independence)

46. Andrejs Šķesters, Riga Stradiņš University, Riga, Latvia

47. Inga Skestere, Latvia

48. Dita Rituma, Riga Stradiņš University, Latvia

49. Prof. Jurate Macijauskiene, ORCID 0000-0001-9343-2010, Lithuanian University of Health Sciences, Mickeviciaus str. 9, LT44307 Kaunas, Lithuania

50. Veronika Matutytė, No ORCID, Lithuanian University of Health Sciences, Mickeviciaus str. 9, LT44307 Kaunas, Lithuania

51. Dr Eleftheria Antoniadou, ORCID 0000-0002-2687-1248, Centre Hospitalier du Nord, 9080 Ettelbruck, Luxembourg

52. Dr Mohamed Salem, ORCID 0009-0006-3966-7656, Active Ageing and Community Care unit, FXB building, Mdina road, QRM9014 Qormi, Malta

53. Ms Francesca Farrugia, 0009-0004-9604-5744, St Vincent de Paul long term care facility, Luqa, Malta

54. Artiom Jucov, ORCID 0000-0003-4664-8323, National Antidoping Agency, 180, Stefan cel Mare blvd., 7 floor MD-2004, State University of Medicine and Pharmacy Nicolae Testemitanu, Chisinau, Moldova

55. Asst. prof. Milena Cojic, ORCID 0000-0002-4395-0626, Family medicine department, Medical faculty, University of Montenegro, 81000 Podgorica, Montenegro

56. Prof. Wilco Achterberg, ORCID 0000-0001-9227-7135, Leiden University Medical Center – LUMC, 2300 RC Leiden, Netherlands

57. Dr Eveline van Poelgeest, ORCID 0000-0002-2021-5602, University Medical Centre Amsterdam, Meibergdreef 9, 1105 AZ Amsterdam, Netherlands

58. Prof. Dr Sonja Genadieva Stavrikj, ORCID 0000-0003-4252-9984, University Hematology Clinic, Medical faculty- Skopje , University Ss Cyril and Methodius - Skopje, Republic of North Macedonia

59. Dr Biljana Petreska-Zovic, ORCID 0000-0003-1734-4478, PHI Specialized Hospital for Geriatric and Palliative Medicine 13 November Skopje, Boris Sarafov 129, 1000 Skopje, Republic of North Macedonia

60. Prof. Anette Hylen Ranhoff, ORCID 0000-0001-8690-4753, University of Bergen, 5021 Bergen, Norway

61. Prof. Jerzy Gąsowski, ORCID 0000-0002-8025-5323, Jagiellonian University Medical College, Faculty of Medicine, 30-688 Kraków, Poland

62. Prof. Zyta Beata Wojszel, ORCID 0000-0002-6472-5241, Department of Geriatrics, Medical University of Bialystok, Poland and Hospital of the Ministry of Interior, Fabryczna str. 27, 15-471 Bialystok, Poland

63. Anna Rudzińska, ORCID 0000-0002-8369-2131, Jagiellonian University Medical College, Kraków, Poland

64. Dr Robert Kupis, ORCID 0000-0003-4594-2061, Jagiellonian University Medical College, Kraków, Poland

65. Dr Ian Perera, ORCID 0000-0002-9269-4155, Department of Internal Medicine and Gerontology, Jagiellonian University Medical College, Kraków, Poland

66. Prof. Mariana Alves, ORCID 0000-0002-1369-8423, Faculda de Medicina Universidade de Lisboa - Faculdade de Medicina Universidade de Lisboa, Av. Prof. Egas Moniz, 1649-028 Lisbon, Portugal

67. Dr Helena Monteiro, ORCID 0000-0003-0626-9556, Serviço Nacional de Saúde, Unidade Local de Saúde Lisboa Ocidental, Cascais, Portugal

68. Dr Ana Farinha, ORCID 0000-0002-5402-7626, Centro Hospitalar de Setubal, 2910-446 Setúbal, Portugal

69. Prof. Ioana Dana Alexa, 0000-0001-5795-4161, University of Medicine and Pharmacy "Gr.T. Popa" Iasi, 700115 Iasi, Romania

70. Prof. Gabriel Prada, ORCID 0000-0002-4762-9581, Department of Geriatric Medicine and Gerontology, “Carol Davila” University of Medicine and Pharmacy Bucharest, Romania

71. Dr Ana-Gabriela Prada, ORCID 0000-0002-5306-4775, “Carol Davila” University of Medicine and Pharmacy, Bucharest, Romania

72. Dr Adina Carmen Ilie, ORCID 0000-0002-9152-8534, Grigore T. Popa University of Medicine and Pharmacy, 700115 Iasi, Romania

73. Associate Prof. Dr Aleksandra Popovac, ORCID 0000-0002-4303-2526, University of Belgrade - University of Belgrade, School of Dental Medicine, 11000 Belgrade, Serbia

74. Dr Slađana Šobajić, ORCID 0000-0002-4047-6121, University of Belgrade-Faculty of Pharmacy - Faculty of Pharmacy, University of Belgrade, 11221 Belgrade, Serbia

75. Prof. Dr Tatjana Puškar, ORCID 0000-0002-7567-5622, Faculty of Medicine Novi Sad, Novi Sad, Serbia

76. Associate Prof. Dr Milica Jeremić Knežević, ORCID 0000-0002-2802-8780, Faculty of Medicine University of Novi Sad, Serbia

77. Danica Sazdanić-Velikić, ORCID 0000-0001-6639-4987, Faculty of Medicine, University of Novi Sad; Department of General Medicine and Geriatrics, Institute for pulmonary diseases of Vojvodina, Sremska Kamenica, Serbia

78. Ana Tomas, ORCID 0000-0003-2361-872X, Department of Pharmacology, Toxicology and Clinical Pharmacology, Faculty of Medicine, University of Novi Sad, Serbia

79. Dr Biljana Lazović, 0000-0001-8683-4759, University Hospital center Zemun, Faculty of Medicine, University of Belgrade, Serbia

80. Dr Stefan Sredojević, ORCID 0000-0002-8584-7088, Department of Periodontology, School of Dental Medicine, University of Belgrade, Serbia

81. Dr Katarina Stefanović, ORCID 0000-0002-1479-5858, Geriatric Department, Clinical Hospital Zvezdara, Belgrade, Serbia

82. Prof. Ctibor Határ, ORCID 0000-0003-4480-9947, Constantine the Philosopher University in Nitra, Drážovská 4, 949 74 Nitra, Slovakia

83. PhDr. Andrea Franta, ORCID 0009-0006-7021-0793, Faculty of Education Constantine the Philosopher University in Nitra Dražovská 4 941 09 Nitra, Slovakia

84. Dr Santiago Cotobal Rodoles, ORCID 0000-0002-2297-4661, Hospital Universitario Severo Ochoa, Leganés (Madrid), Spain and Universidad Alfonso X el sabio, Villanueva de la Cañada (Madrid), Spain

85. Dr Laura Mónica Perez, ORCID 0000-0003-3152-9882, Parc Sanitari Pere Virgili, Av. Vallcarca 30, 08023 Barcelona, Spain

86. Prof. Thomas Münzer, 0000-0003-1891-1171, Geriatrische Klinik St. Gallen, Rorschacherstrasse 94, 9000 St. Gallen, Switzerland

87. Dr Mathias Schlögl, ORCID 0000-0002-6299-8852, Clinic Barmelweid, Department for Geriatric Medicine, Switzerland, Health Longevity Center Zurich, University of Zurich, Switzerland

88. Dr Radhouane Gouiaa, ORCID 0000-0003-0578-1355, Caisse Nationale de Securite Sociale - Policlinique CNSS – Sfax, Tunisia

89. Prof. Gülistan Bahat, ORCID 0000-0001-5343-9795, Istanbul University, Istanbul Medical Faculty , Department of Internal Medicine, Division of Geriatrics, Capa, 34093, Istanbul, Türkiye

90. Assoc. Prof. Mehmet Ali Aslaner, ORCID 0000-0002-7851-7881, Department of Emergency Medicine, Gazi University Faculty of Medicine, Emniyet neighborhood, Yenimahalle, 06560, Ankara, Türkiye

91. Assoc. Prof. Gözde Şengül Ayçiçek, ORCID 0000-0003-0528-8851, Etlik City Hospital Department of Geriatric Medicine 06170 Ankara, Türkiye

92. Assoc. Prof. Nilüfer Demiral Yılmaz, ORCID 0000-0002-1305-3035, Ege University Faculty of Medicine Department of Medical Education, 35100 Izmir, Türkiye

93. Assoc. Prof. Dr Mustafa Said Yıldız, ORCID 0000-0001-7423-1408, Ministy of Health, Türkiye

94. Prof. Gerald McKenna, ORCID 0000-0001-8478-1673, Centre for Public Health, Queen’s University Belfast, Belfast, United Kingdom
