# Supplementary material for: Educational Needs in Geriatric Medicine Among Health Care Professionals and Medical Students in COST Action 21122 PROGRAMMING: Mixed-Methods Survey Protocol
Source: JMIR Res Protoc. 2025 Jun 3;14:e64985. doi: 10.2196/64985 (PMC12174867; doi:10.2196/64985)
Supplement: Multimedia Appendix 10 [file resprot_v14i1e64985_app10.docx]

**Multimedia Appendix 10: Extended proposal form**

**Proposal form for analysis of the PROGRAMMING survey data on educational needs**

**Indicative full title of proposed paper / undergraduate, postgraduate or PhD thesis / policy document**:

**List of co-authors** (name, affiliation(s), country (or countries) and language(s) spoken)

**Person who will take on the main workload of the study ("workload responsible"): name and ORCID number**

**Person who will be responsible for the compliance of the study with scientific and ethical requirements ("supervisor"): name and ORCID number**

**Please, note that the same person can be workload responsible for two proposals at maximum** (in case the workload responsible is alone, the First Author can still submit the proposal and the PROGRAMMING Review Committee will help find suitable co-authors)

(In any case, the Review Committee may propose potential co-authors to join the authors’ team, according to their scientific expertise and research experience)

**Is this paper / thesis / policy document closely related to the tasks of a Working Group? Please, reply YES or NO; if YES, please, specify the WG**

**Research question**:

**Brief background or rationale**:

**Suggested methods**:

Potential list of questions of the survey of which to retrieve the responses

**Expected novelty/impact**:

**Cover letter**: Submit a brief **cover letter** outlining your clinical, and/or research expertise on the topic of your proposal and the intended research methods, as well as your previous experience in multicentre collaborations.

**Timeline**:

**Language** in which the paper or policy document will be written:

**Is the expertise of a statistician present?**

When applying to analyse the data, please adhere to the following rules.

1. **Acknowledge funding: Please acknowledge the funding** received from the COST Action PROGRAMMING CA21122 in all scientific publications. Use the following statement: “COST Action PROGRAMMING CA21122 has received funding from the European Cooperation in Science and Technology under grant agreement N° AGA-CA21122-1-15351”. Also refer to the Annotated Rules for COST Actions (https://www.cost.eu/uploads/2022/12/COST-094-21-Annotated-Rules-for-COST-Actions-Level-C-V1.3.pdf).
2. **Authorship requirements**: If analysing data from more than one country, please include **at least three different countries**. This is not required in case you are analysing data solely from your own country.
3. **Proposal submission limit**: You may submit a maximum **of two proposals as a ‘workload responsible’ person at any given time**.
4. **Please, kindly submit the final draft of scientific articles to the Review Committee for approval**.
5. Share your work: Please, ensure that your scientific publication, undergraduate, postgraduate or PhD thesis, or policy document is made available to the **Science Communication Coordinator** of the COST Action PROGRAMMING CA21122.

**□ I declare that I will acknowledge COST funding in any scientific publication, undergraduate, postgraduate or PhD thesis, or policy document using the following statement:**  “COST Action PROGRAMMING CA21122 has received funding from the European Cooperation in Science and Technology under grant agreement N° AGA-CA21122-1-15351”

Please, refer to the Annotated Rules for COST Actions (https://www.cost.eu/uploads/2022/12/COST-094-21-Annotated-Rules-for-COST-Actions-Level-C-V1.3.pdf ), in order to acknowledge funding in any scientific publications, presentations or policy documents. In particular, please, refer to A3-3.2 Acknowledging COST and EU Funding for further information. Elements for Acknowledgements include: COST logotype; the EU emblem and text “Funded by the European Union”; the acknowledgement text needs to include the title of the Action (or Acronym) AND the COST Action number; the bollerplate featuring a description of COST; a reference to the COST website.

**□ I declare that I will aim to publish my scientific publication in reputable, renowned, peer-reviewed journals.**

Please, refer to the Annotated Rules for COST Actions. "Recommendation. In selecting the publisher for a scientific publication to be published in Open Access, the Action MC shall select a renowned publisher and the publication shall be of high impact. Impact of the publication to be assessed along the SCImago Journal Rank (SJR)3 – that has established itself as a good alternative to the Impact Factor – of the considered academic journal. Any journal that is situated in the first three quartiles Q1-Q3 is considered of sufficient impact, while journals in the last quartile Q4 or journals not contained in the SCOPUS database need an assessment by the responsible Science Officer; the SJR and quartile (Q1, Q2, Q3 or Q4) for each journal contained in SCOPUS can be obtained from the SCImago webpage (accessible at https://www.scimagojr.com/journalrank.php).

The scientific publication shall be subjected to peer review. Peer review is defined as obtaining advice on individual manuscripts from reviewers, expert in the field who are not part of the journal’s editorial staff. This process, as well as any policies related to the journal’s peer review procedures, shall be clearly described on the journal’s Web site.”

**□ I declare I will submit the final manuscript prior to publication to the RC for approval**

**□ I declare that I will make my abstract and presentation at conference, scientific publication, undergraduate, postgraduate or PhD thesis, or policy document available to the Review Committee and the Science Communication Coordinator of the COST Action PROGRAMMING CA21122.**

**□ I declare that I will not use the retrieved and transferred data for any other purpose outside of the scope of the scientific publication, undergraduate, postgraduate or PhD thesis, or policy document approved by the PROGRAMMING CA21122 Review Committee.**
