# Supplementary material for: Educational Needs in Geriatric Medicine Among Health Care Professionals and Medical Students in COST Action 21122 PROGRAMMING: Mixed-Methods Survey Protocol
Source: JMIR Res Protoc. 2025 Jun 3;14:e64985. doi: 10.2196/64985 (PMC12174867; doi:10.2196/64985)
Supplement: Multimedia Appendix 11 [file resprot_v14i1e64985_app11.docx]

**Multimedia Appendix 11: Simplified proposal form**

**Simplified proposal form for analysis of the PROGRAMMING survey data on educational needs**

**Simplified version for presentation at Conferences only**

**Indicative full title of proposed presentation at Conference**:

**List of co-authors** (name, affiliation(s), country (or countries)

When applicable: Potential list of questions of the WG1 online survey of which to retrieve the responses (the EuGMS secretary and Dr Ogliari are happy to be contacted for discussion on which data are available; emails: [secretary@eugms.org](mailto:secretary@eugms.org) and [Giulia.Ogliari1@nottingham.ac.uk](mailto:Giulia.Ogliari1@nottingham.ac.uk) ) or type of data to retrieve (for Action data besides the online survey).

**Name of Conference**:

**Place and time of Conference**:

**Language** in which the presentation at Conference will be delivered:

**□ I declare that I will acknowledge COST funding in my presentation at Conference using the following statement:**  “COST Action PROGRAMMING CA21122 has received funding from the European Cooperation in Science and Technology under grant agreement N° AGA-CA21122-1-15351”

Please, refer to the Annotated Rules for COST Actions (https://www.cost.eu/uploads/2022/12/COST-094-21-Annotated-Rules-for-COST-Actions-Level-C-V1.3.pdf ), in order to acknowledge funding in any scientific publications, presentations or policy documents. In particular, please, refer to A3-3.2 Acknowledging COST and EU Funding for further information. Elements for Acknowledgements include: COST logotype; the EU emblem and text “Funded by the European Union”; the acknowledgement text needs to include the title of the Action (or Acronym) AND the COST Action number; the bollerplate featuring a description of COST; a reference to the COST website.

**□ I declare that I will make my abstract and presentation at conference available to the Review Committee and the Science Communication Coordinator of the COST Action PROGRAMMING CA21122.**

Every author of an oral presentation or a poster at a Conference is encouraged to submit a paper for publication after the Conference; **in this case, they will have to then complete the Extended proposal form and submit it to the PROGRAMMING Review Committee**.
